# Four 1-aryl-1*H*-pyrazole-3,4-di­carboxyl­ate derivatives: synthesis, mol­ecular conformation and hydrogen bonding

**DOI:** 10.1107/S2056989018015864

**Published:** 2018-11-13

**Authors:** Asma, Balakrishna Kalluraya, Hemmige S. Yathirajan, Ravindranath S. Rathore, Christopher Glidewell

**Affiliations:** a Department of Studies in Chemistry, Mangalore University, Mangalagangotri, Mangalore-574 199, India; b Department of Studies in Chemistry, University of Mysore, Manasagangotri, Mysuru-570 006, India; c Department of Bioinformatics, School of Earth, Biological and Environmental Sciences, Central University of South Bihar, Gaya-824 236, India; d School of Chemistry, University of St Andrews, St Andrews, Fife KY16 9ST, UK

**Keywords:** synthesis, 1,3-dipolar addition, crystal structure, mol­ecular conformation, disorder, hydrogen bonding, supra­molecular assembly

## Abstract

Four 1-aryl-1*H*-pyrazole-3,4-di­carboxyl­ate derivatives, one acid, two esters and a dicarbohydrazide have been synthesized starting from 3-aryl sydnones, and structurally characterized. There is an intra­molecular O—H⋯O hydrogen bond in 1-phenyl-1*H*-pyrazole-3,4-di­carb­oxy­lic acid, C_11_H_8_N_2_O_4_, (I), and the mol­ecules are linked into a three-dimensional framework structure by a combination of O—H⋯O, O—H⋯N, C—H⋯O and C—H⋯π(arene) hydrogen bonds. In each of the two esters dimethyl 1-phenyl-1*H*-pyrazole-3,4-di­carboxyl­ate, C_13_H_12_N_2_O_4_, (II), and dimethyl 1-(4-methyl­phen­yl)-1*H*-pyrazole-3,4-di­carboxyl­ate, C_14_H_14_N_2_O_4_, (III), C—H⋯O hydrogen bonds lead to the formation of cyclic centrosymmetric dimers: in (III), one of the meth­oxy­carbonyl groups is disordered over two sets of atomic sites having occupancies 0.71 (2) and 0.29 (2). An intra­molecular N—H⋯O hydrogen bond is present in the structure of 1-(4-meth­oxy­phen­yl)-1*H*-pyrazole-3,4-dicarbohydrazide, C_12_H_14_N_6_O_3_, (IV), and the mol­ecules are linked into a three-dimensional framework structure by a combination of N—H⋯O, N—H⋯N, N—H⋯π(arene) and C—H⋯O hydrogen bonds. Comparisons are made with the structures of a number of related compounds.

## Abstract 2

1-Phenyl-1*H*-pyrazole-3,4-di­carb­oxy­lic acid and 1-(4-meth­oxy­phen­yl)-1*H*-pyrazole-3,4-dicarbohydrazide form complex hydrogen-bonded framework structures each containing multiple hydrogen-bond types, but dimethyl 1-phenyl-1*H*-pyrazole-3,4-di­carboxyl­ate and dimethyl 1-(4-methyl­phen­yl)-1*H*-pyrazole-3,4-di­carboxyl­ate form simple cyclic dimers containing only C—H⋯O hydrogen bonds.

## Chemical context   

**Figure 1:**
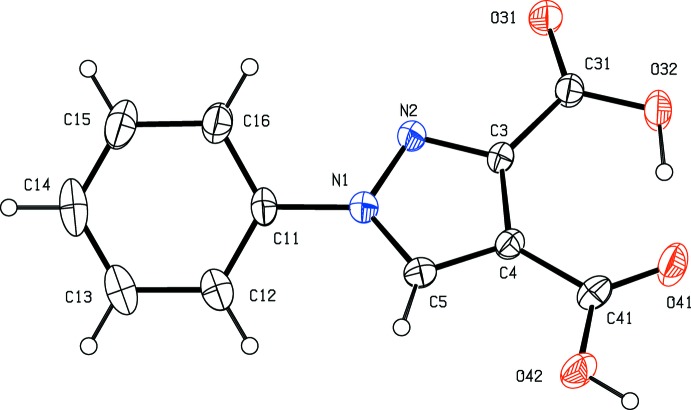
The mol­ecular structure of compound (I) showing the atom-labelling scheme. Displacement ellipsoids are drawn at the 30% probability level.

Pyrazole derivatives have been shown to exhibit a wide range of biological activities including analgesic (Girisha *et al.*, 2010 ▸), anti­convulsant (Owen *et al.*, 1958 ▸), anti­microbial (Satheesha & Kalluraya, 2007 ▸; Asma *et al.*, 2018 ▸), anti­tumour (Park *et al.*, 2005 ▸), and insecticidal and larvicidal activity (Yang *et al.*, 2018 ▸). Pyrazole carb­oxy­lic acids and their derivatives are versatile precursors for the synthesis of numerous substituted analogues (Asma *et al.*, 2018 ▸; Devi *et al.*, 2018 ▸) and, with these considerations in mind, we have now synthesized a series of new pyrazole carboxyl­ate derivatives as inter­mediates for the synthesis of new pharmacologically active products. Here we report the syntheses, and the mol­ecular and supra­molecular structures of four such compounds, namely 1-phenyl-1*H*-pyrazole-3,4-di­carb­oxy­lic acid (I), dimethyl 1-phenyl-1*H*-pyrazole-3,4-di­carboxyl­ate (II), dimethyl 1-(4-methyl­phen­yl)-1*H*-pyrazole-3,4-di­carboxyl­ate (III) and 1-(4-meth­oxy­phen­yl)-1*H*-pyrazole-3,4-dicarbohydrazide (IV) (Figs. 1 ▸–4 ▸ ▸ ▸).
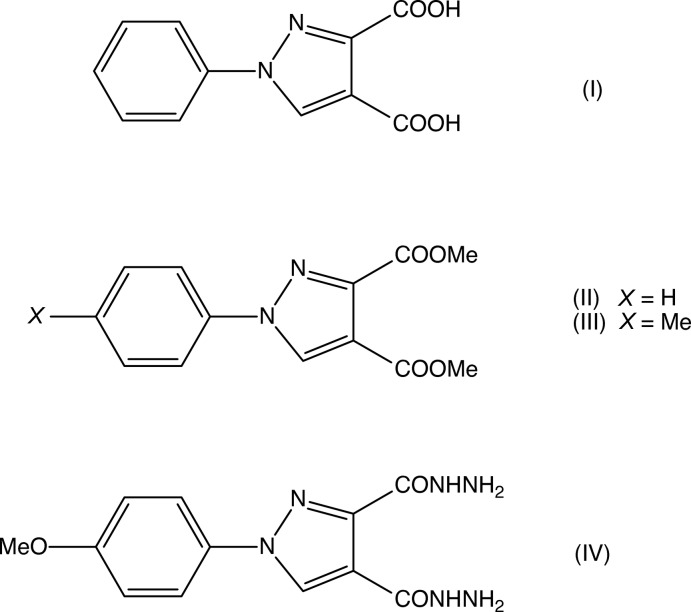


**Figure 5:**
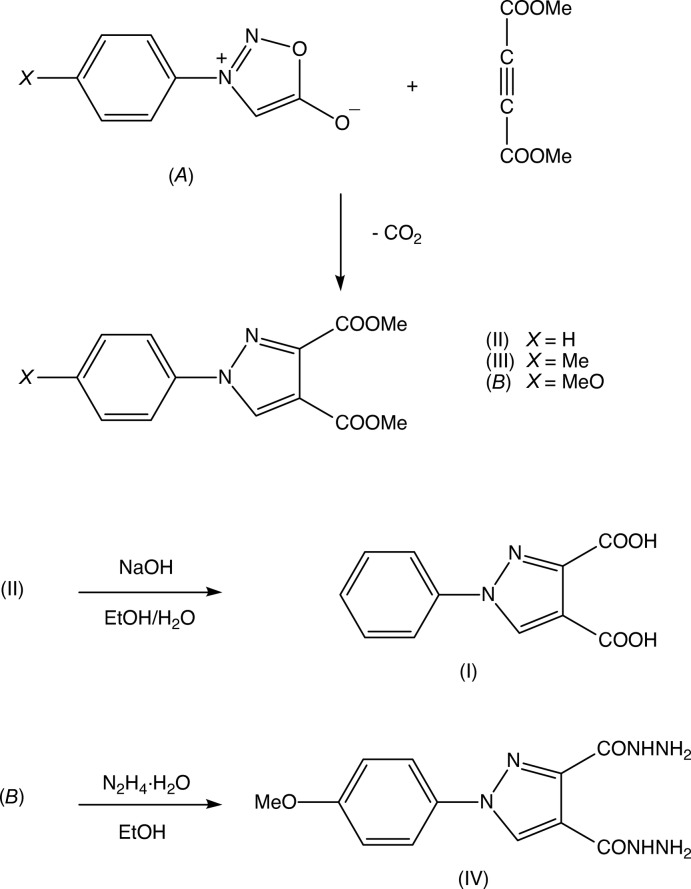
The synthetic routes to compounds (I)–(IV).

The products (II) and (III) and the inter­mediate ester (*B*) (Fig. 5 ▸) used in the formation of compound (IV) were all prepared using the 1,3-dipolar addition reaction between dimethyl acetyl­enedi­carboxyl­ate and the 3-aryl­syndones [3-aryl-1,2,3-oxa­diazol-3-ium-5-olates] (*A*), with loss of carbon dioxide in entropy-driven reactions (Huisgen *et al.*, 1962 ▸) (Fig. 5 ▸). Hydrolysis of the ester (II) gave the di­carb­oxy­lic acid (I), while hydrazinolysis of the ester (*B*) gave the dicarbohyrazide (IV). The sydnone precursors (*A*) were all prepared from the corresponding anilines *via* the substituted *N*-aryl-*N*-nitro­soglycines (Greco *et al.*, 1962 ▸; Fun *et al.*, 2010 ▸).

## Structural commentary   

The bond distances in compounds (I)–(IV) show no unexpected values: all are typical of their types (Allen *et al.*, 1987 ▸). However, the mol­ecular conformations show some inter­esting features. In each of (I) and (IV), the two carb­oxy substituents on the pyrazole ring are nearly coplanar with this ring, as shown by the leading torsional angles (Table 1 ▸): this is almost certainly a consequence of the presence on an intra­molecular O—H⋯O in (I) and an intra­molecular N—H⋯O hydrogen bond in (IV) (Table 2 ▸). In compounds (I) and (III), where such intra­molecular inter­actions are not possible, the carboxyl groups at C3 are by no means coplanar with the pyrazole ring (Table 1 ▸), and in compound (III) the 3-meth­oxy­carbonyl substituent is disordered over two sets of atomic sites having occupancies 0.71 (2) and 0.29 (2) in the crystal selected for data collection: the orientations of the two disorder components are related to one another by a rotation about the C3—C31 bond of approximately 23° (Table 1 ▸). It may be noted here that the ketonic O atom O31 acts as a hydrogen-bond acceptor in each of (I) and (IV), but not in (II) and (III) (Table 2 ▸), and the disorder in (III) may be associated with this.

In each of (I) and (II), the planes of the aryl and pyrazole rings make much larger dihedral angles than these planes do in (II) and (IV) (Table 1 ▸). This may be associated with the cooperative effect in (III) and (IV) of the C—H⋯O hydrogen bonds involving atoms C5 and C12 as donors (Table 2 ▸), whereas no such cooperation is found in the structures of (I) and (II).

## Supra­molecular features   

**Figure 6:**
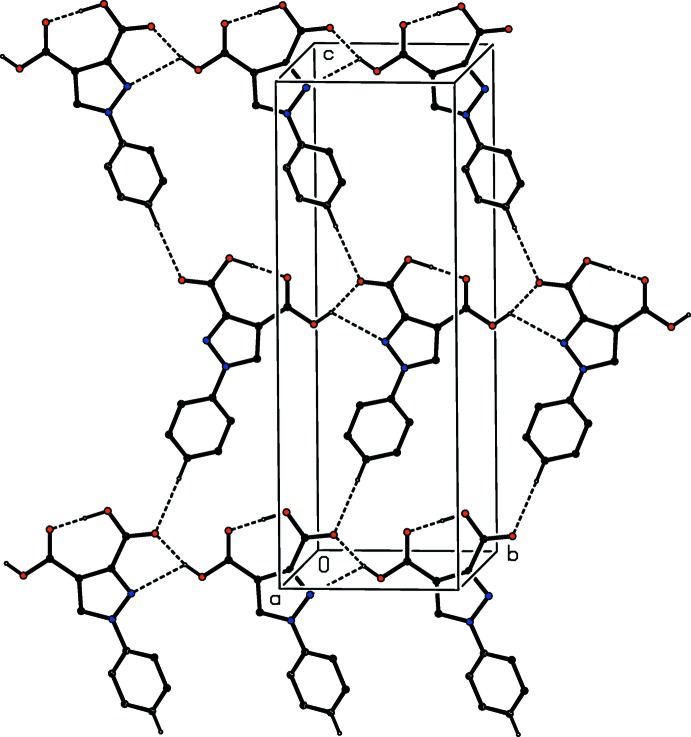
Part of the crystal structure of compound (I) showing the formation of a hydrogen-bonded sheet parallel to (100). Hydrogen bonds are shown as dashed lines and, for the sake of clarity, the H atoms bonded to C atoms but not involved in the motifs shown have been omitted.

The supra­molecular assembly of compound (I) to form a three-dimensional framework structure depends upon four types of hydrogen bonds (Table 2 ▸), and the framework formation can readily be analysed in terms of one-dimensional sub-structures (Ferguson *et al.*, 1998*a* ▸,*b* ▸; Gregson *et al.*, 2000 ▸). A combination of O—H⋯O and O—H⋯N hydrogen bonds, the latter rather weak, links mol­ecules related by translation into a *C*(6)*C*(7)[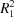
(5)] (Etter, 1990 ▸; Etter *et al.*, 1990 ▸; Bernstein *et al.*, 1995 ▸) chain of rings running parallel to the [010] direction (Fig. 6 ▸). In the second sub-structure, mol­ecules related by the *c*-glide plane at *y* = 0.25 are linked by a C—H⋯O hydrogen bond to form a simple *C*(10) chain running parallel to the [001] direction, and the combination of these two chain motifs generates an almost planar sheet lying parallel to (100) in the domain 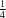 < *x* < 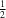 (Fig. 6 ▸). Finally, two weak C—H⋯π(arene) hydrogen bonds link this sheet to the adjacent sheets in the domains 0 < *x* < 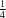 and 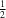 < *x* < 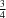, and in this way all of the (100) sheets are linked to form a three-dimensional framework structure.

**Figure 7:**
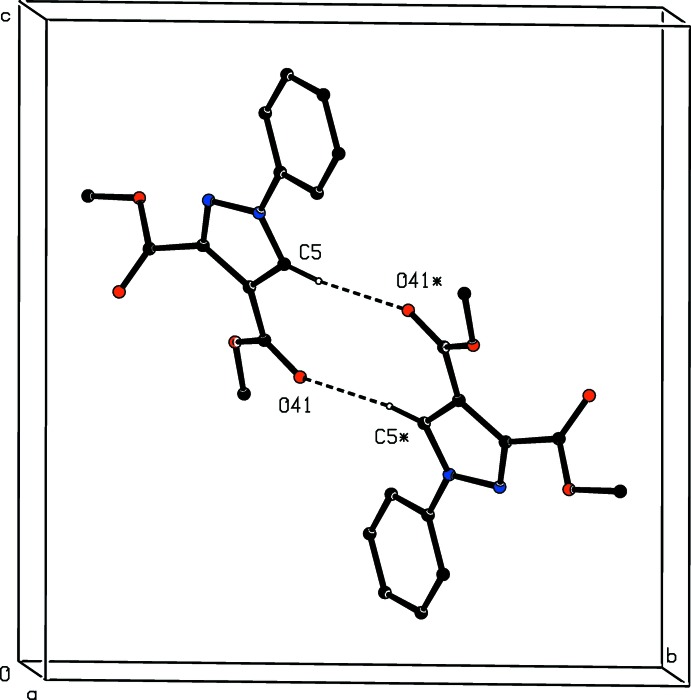
Part of the crystal structure of compound (II) showing the formation of a hydrogen-bonded 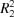
(10) dimer. Hydrogen bonds are shown as dashed lines and, for the sake of clarity, the H atoms not involved in the motif shown have been omitted. The atoms marked with an asterisk (*) are at the symmetry position (1 − *x*, 1 − *y*, 1 − *z*).

**Figure 8:**
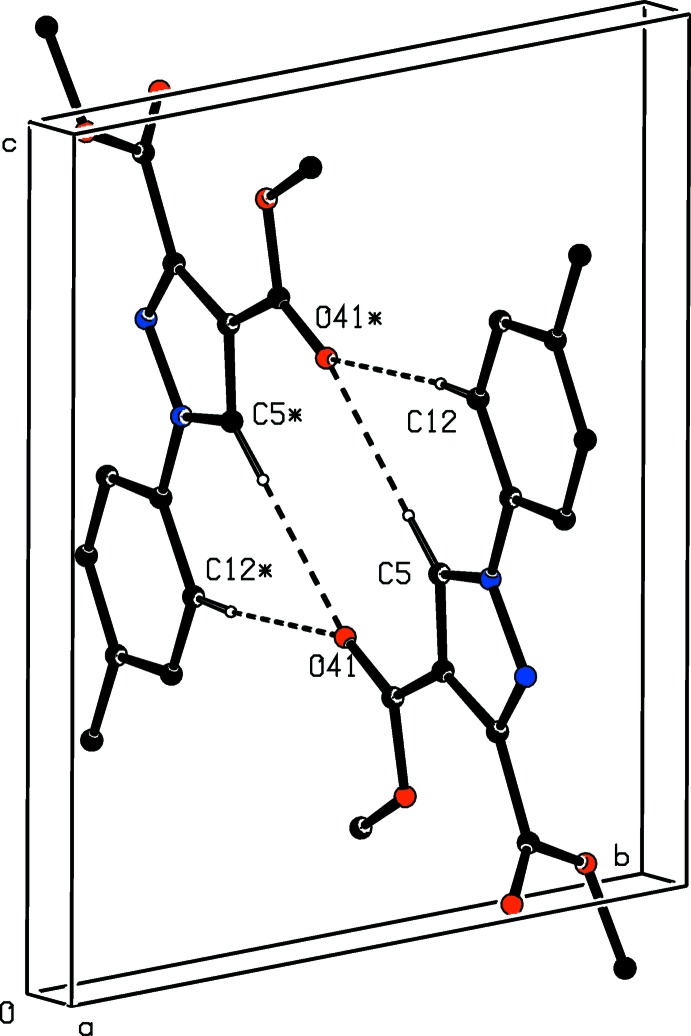
Part of the crystal structure of compound (III) showing the formation of a hydrogen-bonded dimer containing 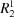
(7), 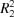
(10) and 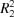
(16) ring motifs. Hydrogen bonds are shown as dashed lines and, for the sake of clarity, the minor disorder component and the H atoms not involved in the motifs shown have been omitted. The atoms marked with an asterisk (*) are at the symmetry position (−*x*, 1 − *y*, 1 − *z*).

By contrast, the supra­molecular assembly in the ester (II) is extremely simple, with inversion-related pairs of mol­ecules linked by C—H⋯O hydrogen bonds (Table 2 ▸) to form a centrosymmetric 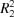
(10) dimer (Fig. 7 ▸). A similar, but more complex centrosymmetric dimer is formed by the ester (III), where the same 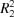
(10) motif as found in (II) is present, along with two flanking 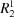
(7) rings within an outer 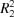
(16) ring (Fig. 8 ▸). In neither (II) nor (III) are there any direction-specific inter­actions between adjacent dimers.

**Figure 9:**
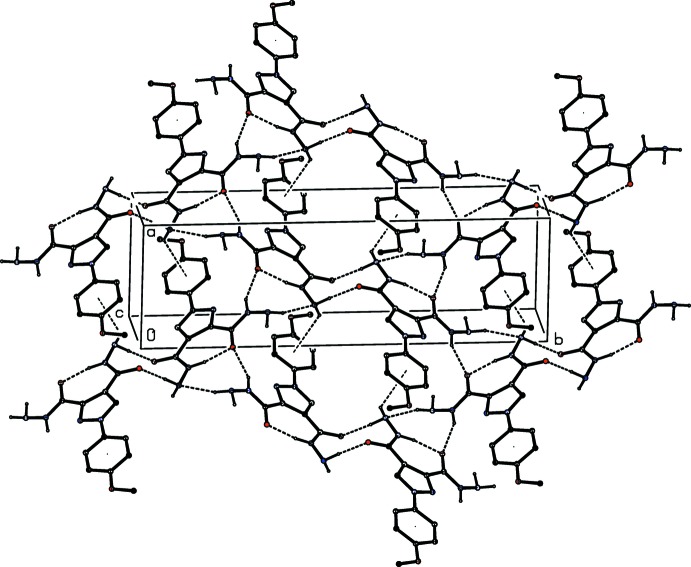
Part of the crystal structure of compound (IV) showing the formation of a hydrogen-bonded sheet lying parallel to (10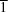) and built from N—H⋯O, N—H⋯N and N—H⋯π(arene) hydrogen bonds. Hydrogen bonds are shown as dashed lines and, for the sake of clarity, the H atoms bonded to C atoms have been omitted.

**Figure 10:**
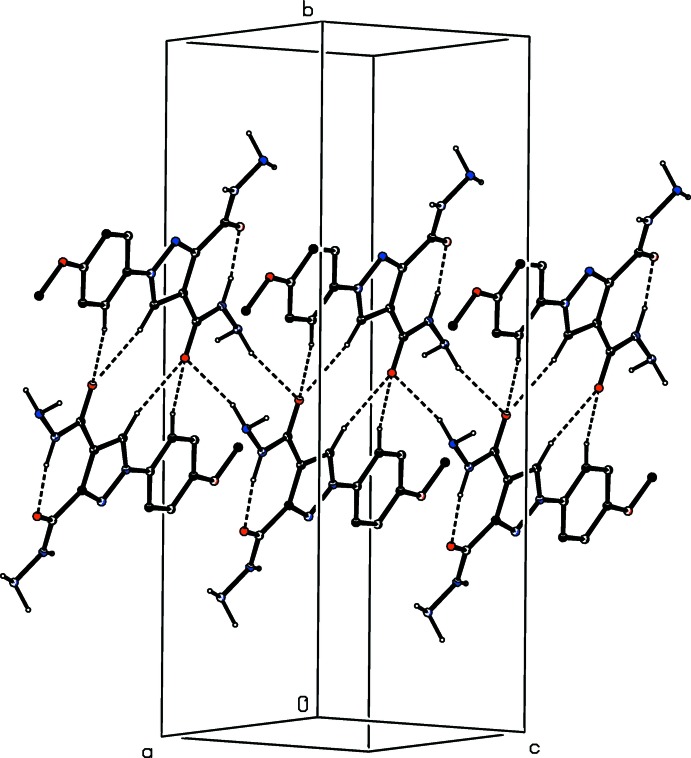
Part of the crystal structure of compound (IV) showing the formation of a hydrogen-bonded chain of rings parallel to [001] and built from N—H⋯O and C—H⋯O hydrogen bonds. Hydrogen bonds are shown as broken lines and, for the sake of clarity, the H atoms bonded to C atoms but not involved in the motifs shown have been omitted.

The supra­molecular assembly in the hydrazide (IV) is the most complex of those reported here. A three-dimensional framework structure is built from four types of hydrogen bonds: N—H⋯O, N—H⋯N, N—H⋯π(arene) and C—H⋯O (Table 2 ▸). As for (I), the assembly is readily analysed in terms of simpler substructures. The hydrogen bond involving atom H42*A* links an inversion-related pair of mol­ecules into an 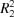
(10) dimer centred at (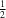, 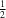, 0), and this finite, zero-dimensional sub-structure can be regarded as the basic building block of the overall structure, which can then be analysed in terms of the ways in which these dimers are linked together. The hydrogen bonds involving the atoms H31 and H32*B* directly link the reference dimer centred at (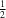, 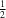, 0) to four similar dimers, centred at (0, 0, −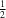), (0, 1, −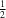), (1, 0, 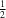) and (1, 1, 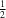), so forming a sheet lying parallel to (10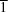) (Fig. 9 ▸), which is reinforced by the N—H⋯π hydrogen bond (Table 2 ▸). The final sub-structure in the assembly of (IV) is one-dimensional: two C—H⋯O hydrogen bonds link the basic 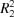
(10) dimers into a chain of rings running parallel to the [001] direction. Within this chain, two types of centrosymmetric 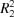
(10) ring can be identified, one containing N—H⋯O hydrogen bonds and the other containing C—H⋯O hydrogen bonds, along with 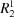
(7) rings (Fig. 10 ▸).

## Database survey   

It is of inter­est to compare briefly the structures of compounds (I)–(IV) reported here with those of some related compounds. In dimethyl 1-(3-chloro-4-meth­yl)-1*H*-pyrazole-3,4-di­carb­oxyl­ate, which differs from (III) only in the presence of the additional 3-chloro substituent, there are again two C—H⋯O hydrogen bonds in the structure, involving exactly the same pair of C—H bonds as in (III), but here the mol­ecules are linked into a *C*(5)*C*(8)[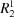
(7)] chain of rings, rather than into cyclic dimers (Thamotharan *et al.*, 2003 ▸). The esters dimethyl 5-(4-chloro­phen­yl)-1-phenyl-1*H*-pyrazole-3,4-di­carboxyl­ate (Li *et al.*, 2014 ▸) and dimethyl 5-(4-bromo­phen­yl)-1-phenyl-1*H*-pyrazole-3,4-di­carboxyl­ate (Alizadeh *et al.*, 2010 ▸), which carry an additional substituent in the pyrazole ring, are isostructural, and the mol­ecules are linked by C—H⋯O hydrogen bonds to form simple chains.

The structures of several esters derived from 1-substituted-1*H*-pyrazole-3,5-di­carb­oxy­lic acids have been reported, including dimethyl 1-(2-cyano­benz­yl)-1*H*-pyrazole-3,5-di­carboxyl­ate (Xiao & Zhao, 2009 ▸), dimethyl 1-(4-cyano­benz­yl)-1*H*-pyrazole-3,5-di­carboxyl­ate (Yao *et al.*, 2009 ▸) and dimethyl 1-cyano­methyl-1*H*-pyrazole-3,5-di­carboxyl­ate (Qu, 2009 ▸). There are no significant inter­molecular inter­actions in either of the benzyl derivatives, but the inversion-related pairs of mol­ecules of the 1-cyano­methyl compound are linked by C—H⋯O hydrogen bonds to form centrosymmetric 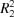
(10) dimers.

**Figure 11:**
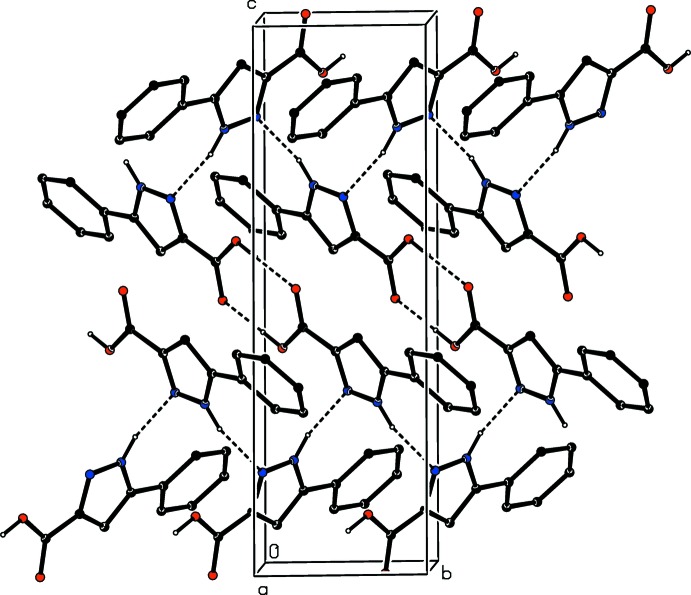
Part of the crystal structure of 3-phenyl-1*H*-pyrazole-5-carb­oxy­lic acid showing the formation of a sheet of 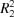
(8) and 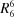
(28) rings lying parallel to (100): hydrogen bonds are shown as dashed lines. The original atomic coordinates (Zhang *et al.*, 2007 ▸) have been used and, for the sake of clarity, the H atoms bonded to C atoms have all been omitted.

In each of 1-benzyl-3-phenyl-1*H*-pyrazole-5-carb­oxy­lic acid (Tang *et al.*, 2007 ▸) and 1-cyclo­hexyl-5-(4-meth­oxy­phen­yl)-1*H*-pyrazole-4-carb­oxy­lic acid (Fun *et al.*, 2011 ▸), inversion-related pairs of mol­ecules are linked by O—H⋯O hydrogen bonds to form centrosymmetric 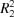
(8) dimers. For the simpler analogue 3-phenyl-1*H*-pyrazole-5-carb­oxy­lic acid, the structure was described (Zhang *et al.*, 2007 ▸) as consisting of chains built from O—H⋯O and N—H⋯N hydrogen bonds, which were then linked into sheets by C—H⋯O hydrogen bonds. However, scrutiny of the atomic coordinates shows that the structure contains no C—H⋯O hydrogen bonds, and that the combin­ation of one O—H⋯O hydrogen bond and one N—H⋯N hydrogen bond generates sheets lying parallel to (100) and containing alternating 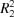
(8) and 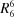
(28) rings (Fig. 11 ▸).

Finally, we note that structures have been reported for each of the precursor sydnones employed here (Fig. 5 ▸), for *X* = H (Hope, 1978 ▸), *X* = Me (Wang *et al.*, 1984 ▸) and *X* = MeO (Fun *et al.*, 2010 ▸) although, when *X* = H, there are no atomic coordinates deposited in the Cambridge Structural Database (Groom *et al.*, 2016 ▸).

## Synthesis and crystallization   

The precursor sydnones (*A*) (Fig. 5 ▸) were prepared from the corresponding anilines (Greco *et al.*, 1962 ▸; Wang *et al.*, 1984 ▸; Fun *et al.*, 2010 ▸). For the synthesis of the esters (II) and (III), a mixture of the sydnone of type (*A*) having *X* = H for (II) or *X* = CH_3_ for (III), (1 mmol) and dimethyl acetyl­enedi­carboxyl­ate (1 mmol) in dry *p*-xylene (10 ml) was heated under reflux for 1 h. The mixtures were then cooled to ambient temperature, the solvent was removed under reduced pressure and the resulting solid products were recrystallized from ethanol. (II): yield 95%, m.p. 373 K. IR (ATR, cm^−1^) 1712 (C=O), 1582 (C=N). NMR (CDCl_3_) δ(^1^H) 3.81 (*s*, 3H, O—CH_3_), 4.08 (*s*, 3H, O—CH_3_), 7.31 (*m*, 1H, H14), 7.40 (*d*, *J* = 7.5 Hz, 2H, H13 & H15), 7.81 (*d*, *J* = 7.5 Hz, 2H, H12 & H16), 9.28 (*s*, 1H, H5). Analysis found C 60.2, H 4.7, N 10.8%, C_13_H_12_N_2_O_4_ requires C 60.0, H 4.6, N 10.8%. (III): yield 93%, m.p. 371 K. IR (ATR, cm^−1^) 1732 (C=O), 1532 (C=N). NMR (CDCl_3_) δ(^1^H) 2.21 (*s*, 3H, C—CH_3_), 3.82 (*s*, 3H, O—CH_3_), 4.10 (*s*, 3H, O—CH_3_), 7.48 (*d*, *J* = 7.6 Hz, 2H, H13 & H15), 7.88 (*d*, *J* = 7.6 Hz, 2H, H12 & H16), 8.94 (*s*, 1H, H5). Analysis found C 61.4, H 5.2, N 10.4%, C_14_H_14_N_2_O_4_ requires C 61.3, H 5.1, N 10.2%.

For the synthesis of the acid (I), the ester (II) (1 mmol) and solid sodium hydroxide (2 mmol) were dissolved in a water–ethanol mixture (water:ethanol 80:20 *v*/*v*, 50 ml). This mixture was heated under reflux for 2h, cooled to ambient temperature and then acidified to pH 2 using dilute aqueous hydro­chloric acid. The resulting solid product was collected by filtration, washed with water and then recrystallized from ethanol. (I): yield 71%, m.p. 508–509 K. IR (ATR, cm^−1^) 3427 (O—H), 1717 (C=O), 1542 (C=N). NMR (CDCl_3_) δ(^1^H) 7.41 (*m*, 1H, H14), 7.53 (*d*, *J* = 7.6 Hz, 2H, H13 & H15), 7.93 (*d*, *J* = 7.6 Hz, 2H, H12 & H16), **?**.10 (*s*, 1H, H5). LC–MS *m*/*z* 230.9. Analysis found C 57.1, H 3.6, N 12.2%, C_11_H_8_N_2_O_4_ requires C 56.9, H 3.5, N 12.1%. For the synthesis of the hydrazide (IV), the inter­mediate ester (*B*) (Fig. 5 ▸) was prepared in exactly the same fashion of the esters (II) and (III), yield 90%, m.p. 458 K. A mixture of ester (*B*) (1 mmol) and hydrazine hydrate (99% aqueous solution, 10 mmol) in ethanol (10 ml) was heated under reflux for 2 h. The mixture was cooled to ambient temperature and the resulting solid product was collected by filtration and then recrystallized from ethanol. (IV): yield 75%, m.p. 502 K. IR (ATR, cm^−1^) 3354 (N—H), 3308 (N—H), 1650 (C=O), 1562 (C=N). NMR (DMSO-*d*_6_) δ(^1^H) 3.67 (*br*, 6H, N-H), 3.82 (*s*, 3H, O-CH_3_), 6.98 (*d*, *J* = 7.7 Hz, 2H, H13 & H15), 7.69 (d, J = 7.7 Hz, 2H, H12 & H16), 8.91 (s, 1H, H5). LC-MS *m*/*z* 290.3. Analysis found C 49.5, H 4.8, N 28.8%, C_12_H_14_N_6_O_3_ requires C 49.6, H 4.9, N 29.0%. Crystals of compounds (I)–(IV) suitable for single-crystal X-ray diffraction were selected directly from the purified samples.

## Refinement   

Crystal data, data collection and structure refinement details are summarized in Table 3 ▸. One low-angle reflection, (001) in compound (III), which had been attenuated by the beam stop was removed from the data set. All H atoms were located in difference maps. The H atoms bonded to C atoms were subsequently treated as riding atoms in geometrically idealized position with C—H distances 0.93 Å (aryl and pyrazole) or 0.96 Å (CH_3_) and with *U*_iso_(H) = *kU*_eq_(C), where *k* = 1.5 for the methyl groups, which were permitted to rotate but not to tilt, and 1.2 for all other H atoms bonded to C atoms. For the H atoms bonded to O or N atoms, the atomic coordinates were refined with *U*_iso_(H) = 1.5*U*_eq_(O) or 1.2*U*_eq_(N), leading to the O—H and N—H distances shown in Table 2 ▸. It was apparent that one of the ester substituents in compound (III) was disordered over two sets of atomic sites. For the minor disorder component, the bonded distances and the 1,2 non-bonded distances were restrained to be the same as the corresponding distances in the major disorder component, subject to s.u. values of 0.005 and 0.01 Å, respectively. In addition, the anisotropic displacement parameters for the corresponding pairs of atoms in the two disorder components were constrained to be the same, and the two disordered carboxyl­ate fragments were constrained to be planar. Subject to these conditions, the occupancies of the two sets of sites refined to 0.71 (2) and 0.29 (2).

## Supplementary Material

Crystal structure: contains datablock(s) global, I, II, III, IV. DOI: 10.1107/S2056989018015864/zl2741sup1.cif

Structure factors: contains datablock(s) I. DOI: 10.1107/S2056989018015864/zl2741Isup2.hkl

Structure factors: contains datablock(s) II. DOI: 10.1107/S2056989018015864/zl2741IIsup3.hkl

Structure factors: contains datablock(s) III. DOI: 10.1107/S2056989018015864/zl2741IIIsup4.hkl

Structure factors: contains datablock(s) IV. DOI: 10.1107/S2056989018015864/zl2741IVsup5.hkl

Supporting information file. DOI: 10.1107/S2056989018015864/zl2741Isup6.cml

Supporting information file. DOI: 10.1107/S2056989018015864/zl2741IIsup7.cml

Supporting information file. DOI: 10.1107/S2056989018015864/zl2741IIIsup8.cml

Supporting information file. DOI: 10.1107/S2056989018015864/zl2741IVsup9.cml

CCDC references: 1877912, 1877911, 1877910, 1877909

Additional supporting information:  crystallographic information; 3D view; checkCIF report

## Figures and Tables

**Figure 2:**
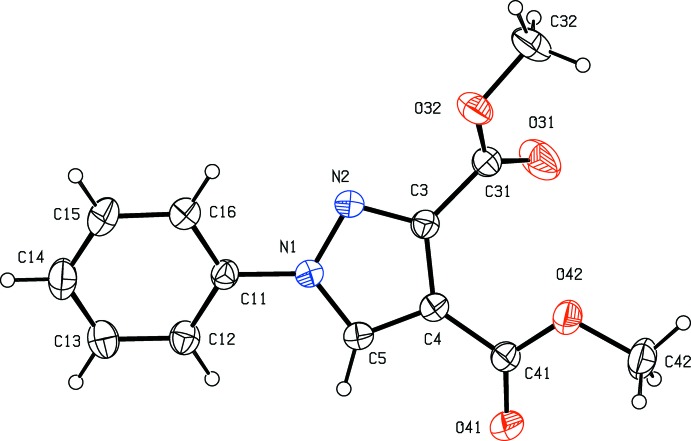
The mol­ecular structure of compound (II) showing the atom-labelling scheme. Displacement ellipsoids are drawn at the 30% probability level.

**Figure 3:**
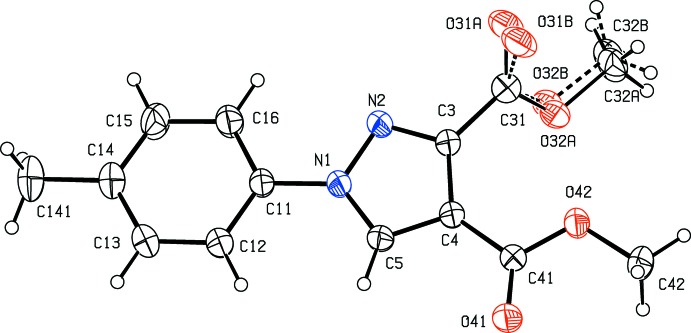
The mol­ecular structure of compound (III) showing the atom-labelling scheme. The major disorder component, occupancy 0.71 (2), is drawn using full lines and the minor component, occupancy 0.29 (2), is drawn using dashed lines. Displacement ellipsoids are drawn at the 30% probability level.

**Figure 4:**
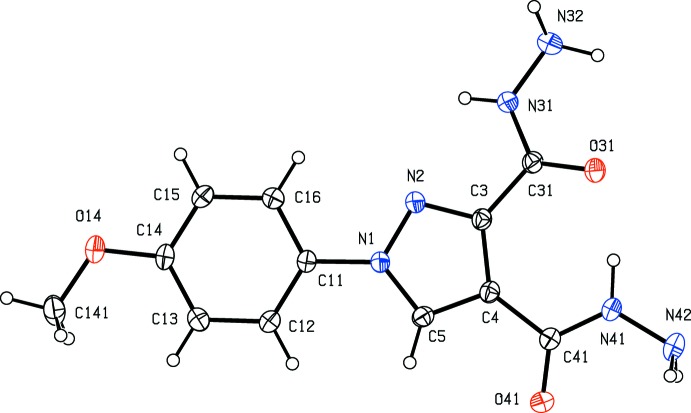
The mol­ecular structure of compound (IV) showing the atom-labelling scheme. Displacement ellipsoids are drawn at the 30% probability level.

**Table 1:** Selected torsional and dihedral angles (°) φ_1_ represents the dihedral angle between the planes of the aryl and pyrazole rings and φ_2_ represents the dihedral angle between the planes (C3,C31,O31*A*,O32*A*) and (C3,C31,O31*B*,O32*B*)

	(I)	(II)	(III)	(IV)
C4—C3—C31—O31	−178.0 (2)	44.8 (3)		−12.5 (4)
C4—C3—C31—O32	2.1 (4)	−135.9 (2)		
C4—C3—C31—O31*A*			−129.1 (9)	
C4—C3—C31—O31*B*			−96.6 (9)	
C4—C3—C31—O32*A*			57.5 (6)	
C4—C3—C31—O32*B*			71.6 (8)	
C4—C3—C31—N31				168.4 (2)
C3—C4—C41—O41	−2.5 (4)	−168.5 (2)	176.5 (2)	−169.0 (2)
C3—C4—C41—O42	178.0 (2)	12.8 (3)	−3.0 (3)	
C3—C4—C41—N41				9.8 (4)
φ_1_	29.38 (8)	24.38 (12)	2.78 (12)	5.82 (13)
φ_2_			22.7 (5)	

**Table 2:** Hydrogen bonds and short inter­molecular contacts (Å, °) *Cg*1 represents the centroid of the C11–C16 ring.

Compound	*D*—H⋯*A*	*D*—H	H⋯*A*	*D*⋯*A*	*D*—H⋯*A*
(I)	O32—H32⋯O41	1.00 (3)	1.54 (3)	3.546 (2)	178 (2)
	O42—H42⋯O31^i^	0.88 (3)	1.80 (3)	2.660 (2)	168 (3)
	O42—H42⋯N2^i^	0.88 (3)	2.56 (3)	3.063 (3)	117 (2)
	C14—H14⋯O31^ii^	0.93	2.53	3.456 (3)	177
	C12—H12⋯*Cg*1^iii^	0.93	2.86	3.685 (3)	148
	C15—H15⋯*Cg*1^iv^	0.93	2.92	3.755 (3)	151
(II)	C5—H5⋯O41^v^	0.93	2.41	3.331 (3)	170
(III)	C5—H5⋯O41^vi^	0.93	2.33	3.249 (3)	168
	C12—H12⋯O41^vi^	0.93	2.43	3.352 (3)	173
(IV)	N31—H31⋯O31^vii^	0.88 (2)	2.04 (2)	2.851 (3)	153 (2)
	N32—H32*A*⋯O14^viii^	0.97 (3)	2.58 (3)	3.256 (3)	127 (2)
	N32—H32*B*⋯N42^vii^	1.00 (2)	2.34 (3)	3.317 (3)	165 (2)
	N41—H41⋯O31	0.95 (3)	1.78 (3)	2.714 (3)	166 (2)
	N42—H42*A*⋯O41^ix^	0.95 (3)	2.21 (3)	3.120 (3)	162 (2)
	N42—H42*B*⋯*Cg*1^*x*^	0.83 (3)	2.85 (3)	3.442 (3)	130 (2)
	C5—H5⋯O41^v^	0.93	2.40	3.314 (3)	166
	C12—H12⋯O41^v^	0.93	2.44	3.354 (3)	168

Symmetry codes: (i) *x*, 1 + *y*, *z*; (ii) *x*, 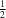 − *y*, −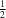 + *z*; (iii) 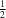 − *x*, 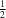 + *y*, *z*; (iv) 1 − *x*, −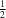 + *y*, 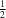 − *z*; (v) 1 − *x*, 1 − *y*, 1 − *z*; (vi) −*x*, 1 − *y*, 1 − *z*; (vii) 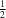 + *x*, 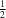 − *y*, 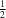 + *z*; (viii) −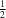 + *x*, 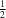 − *y*, −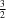 + *z*; (ix) 1 − *x*, 1 − *y*, −*z*; (*x*) −1 + *x*, *y*, −1 + *z*.

**Table 3:** Experimental details

	(I)	(II)	(III)	(IV)
Crystal data				
Chemical formula	C_11_H_8_N_2_O_4_	C_13_H_12_N_2_O_4_	C_14_H_14_N_2_O_4_	C_12_H_14_N_6_O_3_
*M*_r_	232.19	260.25	274.27	290.29
Crystal system, space group	Orthorhombic, *P* *b* *c* *a*	Monoclinic, *P*2_1_/*n*	Triclinic, *P* 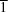	Monoclinic, *P*2_1_/*n*
Temperature (K)	296	296	296	296
*a*, *b*, *c* (Å)	13.164 (2), 7.4692 (9), 21.173 (3)	5.9000 (4), 14.5273 (12), 14.8726 (12)	7.6546 (5), 8.0959 (5), 11.3065 (6)	7.6030 (6), 22.6605 (19), 7.6751 (7)
α, β, γ (°)	90, 90, 90	90, 98.867 (3), 90	78.988 (3), 85.527 (3), 87.548 (4)	90, 102.284 (3), 90
*V* (Å^3^)	2081.8 (6)	1259.51 (17)	685.40 (7)	1292.05 (19)
*Z*	8	4	2	4
Radiation type	Mo *K*α	Mo *K*α	Mo *K*α	Mo *K*α
μ (mm^−1^)	0.12	0.10	0.10	0.11
Crystal size (mm)	0.16 × 0.14 × 0.11	0.17 × 0.14 × 0.13	0.16 × 0.15 × 0.12	0.14 × 0.13 × 0.11

Data collection				
Diffractometer	Bruker Kappa APEXII CCD	Bruker Kappa APEXII CCD	Bruker Kappa APEXII CCD	Bruker Kappa APEXII CCD
Absorption correction	Multi-scan (*SADABS*; Sheldrick, 2008*a* ▸)	Multi-scan (*SADABS*; Sheldrick, 2008*a* ▸)	Multi-scan (*SADABS*; Sheldrick, 2008*a* ▸)	Multi-scan (*SADABS*; Sheldrick, 2008*a* ▸)
*T*_min_, *T*_max_	0.931, 0.987	0.960, 0.987	0.955, 0.988	0.929, 0.988
No. of measured, independent and observed [*I* > 2σ(*I*)] reflections	31526, 2218, 1304	21653, 2704, 1624	12818, 2526, 1777	20456, 2521, 1722
*R*_int_	0.063	0.046	0.030	0.053
(sin θ/λ)_max_ (Å^−1^)	0.634	0.635	0.605	0.618

Refinement				
*R*[*F* ^2^ > 2σ(*F* ^2^)], *wR*(*F* ^2^), *S*	0.045, 0.122, 1.03	0.042, 0.129, 1.04	0.044, 0.128, 1.05	0.046, 0.108, 1.05
No. of reflections	2218	2704	2526	2521
No. of parameters	161	175	196	210
No. of restraints	0	0	7	0
H-atom treatment	H atoms treated by a mixture of independent and constrained refinement	H-atom parameters constrained	H-atom parameters constrained	H atoms treated by a mixture of independent and constrained refinement
Δρ_max_, Δρ_min_ (e Å^−3^)	0.19, −0.16	0.19, −0.18	0.24, −0.24	0.20, −0.21

Computer programs: *APEX2*, *SAINT* and *XPREP* (Bruker, 2004 ▸), *SHELXS97* (Sheldrick, 2008*b* ▸), *SHELXL2014* (Sheldrick, 2015 ▸) and *PLATON* (Spek, 2009 ▸).

## supplementary crystallographic information

### 1-Phenyl-1H-pyrazole-3,4-dicarboxylic acid (I) Crystal data

**Table:**

C_11_H_8_N_2_O_4_	*D*_x_ = 1.482 Mg m^−^^3^
*M**_r_* = 232.19	Mo *K*α radiation, λ = 0.71073 Å
Orthorhombic, *P**b**c**a*	Cell parameters from 2218 reflections
*a* = 13.164 (2) Å	θ = 2.5–26.8°
*b* = 7.4692 (9) Å	µ = 0.12 mm^−^^1^
*c* = 21.173 (3) Å	*T* = 296 K
*V* = 2081.8 (6) Å^3^	Block, colourless
*Z* = 8	0.16 × 0.14 × 0.11 mm
*F*(000) = 960	

### 1-Phenyl-1H-pyrazole-3,4-dicarboxylic acid (I) Data collection

**Table:**

Bruker Kappa APEXII CCD diffractometer	2218 independent reflections
Radiation source: fine focus sealed tube	1304 reflections with *I* > 2σ(*I*)
Graphite monochromator	*R*_int_ = 0.063
φ and ω scans	θ_max_ = 26.8°, θ_min_ = 2.5°
Absorption correction: multi-scan (SADABS; Sheldrick, 2008a)	*h* = −16→16
*T*_min_ = 0.931, *T*_max_ = 0.987	*k* = −9→9
31526 measured reflections	*l* = −26→26

### 1-Phenyl-1H-pyrazole-3,4-dicarboxylic acid (I) Refinement

**Table:**

Refinement on *F*^2^	Hydrogen site location: mixed
Least-squares matrix: full	H atoms treated by a mixture of independent and constrained refinement
*R*[*F*^2^ > 2σ(*F*^2^)] = 0.045	*w* = 1/[σ^2^(*F*_o_^2^) + (0.043*P*)^2^ + 0.9866*P*] where *P* = (*F*_o_^2^ + 2*F*_c_^2^)/3
*wR*(*F*^2^) = 0.122	(Δ/σ)_max_ < 0.001
*S* = 1.03	Δρ_max_ = 0.19 e Å^−^^3^
2218 reflections	Δρ_min_ = −0.16 e Å^−^^3^
161 parameters	Extinction correction: SHELXL2014 (Sheldrick, 2015), Fc^*^=kFc[1+0.001xFc^2^λ^3^/sin(2θ)]^-1/4^
0 restraints	Extinction coefficient: 0.0015 (3)

### 1-Phenyl-1H-pyrazole-3,4-dicarboxylic acid (I) Special details

**Table:**

Geometry. All esds (except the esd in the dihedral angle between two l.s. planes) are estimated using the full covariance matrix. The cell esds are taken into account individually in the estimation of esds in distances, angles and torsion angles; correlations between esds in cell parameters are only used when they are defined by crystal symmetry. An approximate (isotropic) treatment of cell esds is used for estimating esds involving l.s. planes.

### 1-Phenyl-1H-pyrazole-3,4-dicarboxylic acid (I) Fractional atomic coordinates and isotropic or equivalent isotropic displacement parameters (Å^2^)

**Table:**

	*x*	*y*	*z*	*U*_iso_*/*U*_eq_	
N1	0.37444 (14)	0.5732 (2)	0.38946 (7)	0.0382 (4)	
N2	0.37411 (13)	0.4662 (2)	0.44062 (7)	0.0361 (4)	
C3	0.37378 (16)	0.5767 (2)	0.48951 (9)	0.0318 (4)	
C4	0.37462 (16)	0.7576 (2)	0.46953 (10)	0.0341 (5)	
C5	0.37519 (17)	0.7475 (3)	0.40517 (10)	0.0416 (5)	
H5	0.3760	0.8437	0.3773	0.050*	
C11	0.37392 (17)	0.4958 (3)	0.32759 (9)	0.0415 (5)	
C12	0.3296 (2)	0.5877 (4)	0.27889 (11)	0.0572 (7)	
H12	0.2988	0.6978	0.2863	0.069*	
C13	0.3311 (2)	0.5164 (4)	0.21895 (11)	0.0691 (8)	
H13	0.3017	0.5787	0.1856	0.083*	
C14	0.3756 (2)	0.3540 (5)	0.20857 (13)	0.0718 (9)	
H14	0.3771	0.3063	0.1680	0.086*	
C15	0.4181 (2)	0.2612 (4)	0.25766 (13)	0.0665 (8)	
H15	0.4474	0.1499	0.2503	0.080*	
C16	0.41776 (19)	0.3311 (3)	0.31810 (12)	0.0548 (7)	
H16	0.4465	0.2682	0.3515	0.066*	
C31	0.37166 (17)	0.4926 (3)	0.55265 (9)	0.0377 (5)	
O31	0.37408 (14)	0.33081 (19)	0.55896 (7)	0.0540 (5)	
O32	0.36707 (14)	0.5958 (2)	0.60230 (7)	0.0524 (5)	
H32	0.3671 (19)	0.724 (4)	0.5880 (13)	0.079*	
C41	0.37522 (17)	0.9202 (3)	0.50785 (11)	0.0412 (5)	
O41	0.37173 (14)	0.91999 (19)	0.56526 (8)	0.0578 (5)	
O42	0.37916 (14)	1.06752 (19)	0.47441 (8)	0.0528 (5)	
H42	0.375 (2)	1.164 (4)	0.4979 (13)	0.079*	

### 1-Phenyl-1H-pyrazole-3,4-dicarboxylic acid (I) Atomic displacement parameters (Å^2^)

**Table:**

	*U*^11^	*U*^22^	*U*^33^	*U*^12^	*U*^13^	*U*^23^
N1	0.0500 (11)	0.0337 (9)	0.0309 (9)	0.0033 (9)	−0.0029 (8)	−0.0027 (7)
N2	0.0487 (11)	0.0296 (9)	0.0301 (9)	0.0015 (8)	−0.0021 (9)	−0.0017 (7)
C3	0.0375 (11)	0.0287 (9)	0.0293 (10)	0.0002 (9)	−0.0010 (9)	−0.0059 (8)
C4	0.0404 (12)	0.0279 (10)	0.0340 (12)	0.0011 (10)	0.0015 (11)	−0.0031 (8)
C5	0.0562 (14)	0.0302 (11)	0.0383 (12)	0.0037 (10)	−0.0012 (12)	0.0011 (9)
C11	0.0487 (14)	0.0483 (12)	0.0276 (11)	−0.0013 (12)	−0.0019 (10)	−0.0099 (10)
C12	0.0711 (18)	0.0649 (16)	0.0356 (13)	0.0016 (14)	−0.0049 (12)	−0.0026 (12)
C13	0.083 (2)	0.092 (2)	0.0323 (14)	−0.0064 (18)	−0.0068 (13)	−0.0047 (14)
C14	0.074 (2)	0.107 (2)	0.0348 (14)	−0.0165 (19)	0.0040 (15)	−0.0250 (15)
C15	0.0658 (18)	0.0745 (18)	0.0591 (18)	0.0048 (16)	0.0012 (15)	−0.0330 (15)
C16	0.0606 (16)	0.0594 (16)	0.0445 (14)	0.0108 (13)	−0.0067 (13)	−0.0193 (12)
C31	0.0493 (13)	0.0329 (11)	0.0309 (11)	−0.0019 (10)	0.0021 (11)	−0.0031 (9)
O31	0.0923 (13)	0.0305 (8)	0.0392 (9)	0.0023 (9)	0.0035 (9)	0.0028 (7)
O32	0.0871 (13)	0.0404 (9)	0.0296 (8)	−0.0052 (9)	0.0042 (8)	−0.0071 (7)
C41	0.0431 (13)	0.0297 (11)	0.0507 (14)	−0.0011 (10)	0.0061 (11)	−0.0054 (10)
O41	0.0945 (14)	0.0358 (9)	0.0432 (10)	−0.0070 (9)	0.0135 (9)	−0.0126 (7)
O42	0.0783 (13)	0.0255 (8)	0.0547 (11)	0.0011 (8)	0.0056 (9)	−0.0049 (7)

### 1-Phenyl-1H-pyrazole-3,4-dicarboxylic acid (I) Geometric parameters (Å, º)

**Table:**

N1—C5	1.344 (2)	C13—C14	1.365 (4)
N1—N2	1.346 (2)	C13—H13	0.9300
N1—C11	1.432 (2)	C14—C15	1.369 (4)
N2—C3	1.324 (2)	C14—H14	0.9300
C3—C4	1.416 (3)	C15—C16	1.382 (3)
C3—C31	1.477 (3)	C15—H15	0.9300
C4—C5	1.365 (3)	C16—H16	0.9300
C4—C41	1.461 (3)	C31—O31	1.216 (2)
C5—H5	0.9300	C31—O32	1.305 (2)
C11—C12	1.369 (3)	O32—H32	1.00 (3)
C11—C16	1.374 (3)	C41—O41	1.216 (3)
C12—C13	1.377 (3)	C41—O42	1.309 (2)
C12—H12	0.9300	O42—H42	0.88 (3)
			
C5—N1—N2	112.07 (16)	C14—C13—H13	120.1
C5—N1—C11	128.16 (18)	C12—C13—H13	120.1
N2—N1—C11	119.77 (17)	C13—C14—C15	120.2 (2)
C3—N2—N1	105.03 (15)	C13—C14—H14	119.9
N2—C3—C4	111.17 (17)	C15—C14—H14	119.9
N2—C3—C31	116.28 (16)	C14—C15—C16	120.7 (3)
C4—C3—C31	132.55 (17)	C14—C15—H15	119.7
C5—C4—C3	104.21 (16)	C16—C15—H15	119.7
C5—C4—C41	126.91 (18)	C11—C16—C15	118.4 (2)
C3—C4—C41	128.87 (19)	C11—C16—H16	120.8
N1—C5—C4	107.50 (18)	C15—C16—H16	120.8
N1—C5—H5	126.2	O31—C31—O32	119.95 (19)
C4—C5—H5	126.2	O31—C31—C3	121.43 (18)
C12—C11—C16	121.2 (2)	O32—C31—C3	118.62 (17)
C12—C11—N1	119.2 (2)	C31—O32—H32	108.6 (15)
C16—C11—N1	119.6 (2)	O41—C41—O42	122.89 (19)
C11—C12—C13	119.6 (3)	O41—C41—C4	123.62 (19)
C11—C12—H12	120.2	O42—C41—C4	113.48 (19)
C13—C12—H12	120.2	C41—O42—H42	112.5 (18)
C14—C13—C12	119.9 (3)		
			
C5—N1—N2—C3	0.5 (2)	C16—C11—C12—C13	−1.6 (4)
C11—N1—N2—C3	−179.43 (19)	N1—C11—C12—C13	178.4 (2)
N1—N2—C3—C4	−0.4 (2)	C11—C12—C13—C14	0.6 (4)
N1—N2—C3—C31	179.03 (18)	C12—C13—C14—C15	0.6 (4)
N2—C3—C4—C5	0.2 (3)	C13—C14—C15—C16	−0.9 (4)
C31—C3—C4—C5	−179.2 (2)	C12—C11—C16—C15	1.3 (4)
N2—C3—C4—C41	−179.6 (2)	N1—C11—C16—C15	−178.7 (2)
C31—C3—C4—C41	1.1 (4)	C14—C15—C16—C11	−0.1 (4)
N2—N1—C5—C4	−0.4 (3)	N2—C3—C31—O31	2.7 (3)
C11—N1—C5—C4	179.5 (2)	C4—C3—C31—O31	−178.0 (2)
C3—C4—C5—N1	0.2 (3)	N2—C3—C31—O32	−177.2 (2)
C41—C4—C5—N1	179.9 (2)	C4—C3—C31—O32	2.1 (4)
C5—N1—C11—C12	−29.3 (4)	C5—C4—C41—O41	177.8 (2)
N2—N1—C11—C12	150.6 (2)	C3—C4—C41—O41	−2.5 (4)
C5—N1—C11—C16	150.6 (2)	C5—C4—C41—O42	−1.7 (3)
N2—N1—C11—C16	−29.4 (3)	C3—C4—C41—O42	178.0 (2)

### 1-Phenyl-1H-pyrazole-3,4-dicarboxylic acid (I) Hydrogen-bond geometry (Å, º)

**Table:**

*D*—H···*A*	*D*—H	H···*A*	*D*···*A*	*D*—H···*A*
O32—H32···O41	1.00 (3)	1.54 (3)	2.546 (2)	178 (2)
O42—H42···O31^i^	0.88 (3)	1.80 (3)	2.660 (2)	168 (3)
O42—H42···N2^i^	0.88 (3)	2.56 (3)	3.063 (2)	117 (2)
C14—H14···O31^ii^	0.93	2.53	3.456 (3)	177
C12—H12···*Cg*1^iii^	0.93	2.86	3.685 (3)	148
C15—H15···*Cg*1^iv^	0.93	2.92	3.755 (3)	151

Symmetry codes: (i) *x*, *y*+1, *z*; (ii) *x*, −*y*+1/2, *z*−1/2; (iii) −*x*+1/2, *y*+1/2, *z*; (iv) −*x*+1, *y*+1/2, −*z*+1/2.

### Dimethyl 1-phenyl-1H-pyrazole-3,4-dicarboxylate (II) Crystal data

**Table:**

C_13_H_12_N_2_O_4_	*F*(000) = 544
*M**_r_* = 260.25	*D*_x_ = 1.372 Mg m^−^^3^
Monoclinic, *P*2_1_/*n*	Mo *K*α radiation, λ = 0.71073 Å
*a* = 5.9000 (4) Å	Cell parameters from 2704 reflections
*b* = 14.5273 (12) Å	θ = 2.8–26.8°
*c* = 14.8726 (12) Å	µ = 0.10 mm^−^^1^
β = 98.867 (3)°	*T* = 296 K
*V* = 1259.51 (17) Å^3^	Block, orange
*Z* = 4	0.17 × 0.14 × 0.13 mm

### Dimethyl 1-phenyl-1H-pyrazole-3,4-dicarboxylate (II) Data collection

**Table:**

Bruker Kappa APEXII CCD diffractometer	2704 independent reflections
Radiation source: fine focus sealed tube	1624 reflections with *I* > 2σ(*I*)
Graphite monochromator	*R*_int_ = 0.046
φ and ω scans	θ_max_ = 26.8°, θ_min_ = 2.8°
Absorption correction: multi-scan (SADABS; Sheldrick, 2008a)	*h* = −6→7
*T*_min_ = 0.960, *T*_max_ = 0.987	*k* = −18→18
21653 measured reflections	*l* = −18→18

### Dimethyl 1-phenyl-1H-pyrazole-3,4-dicarboxylate (II) Refinement

**Table:**

Refinement on *F*^2^	Hydrogen site location: inferred from neighbouring sites
Least-squares matrix: full	H-atom parameters constrained
*R*[*F*^2^ > 2σ(*F*^2^)] = 0.042	*w* = 1/[σ^2^(*F*_o_^2^) + (0.0511*P*)^2^ + 0.3996*P*] where *P* = (*F*_o_^2^ + 2*F*_c_^2^)/3
*wR*(*F*^2^) = 0.129	(Δ/σ)_max_ < 0.001
*S* = 1.04	Δρ_max_ = 0.19 e Å^−^^3^
2704 reflections	Δρ_min_ = −0.18 e Å^−^^3^
175 parameters	Extinction correction: SHELXL2014 (Sheldrick, 2015), Fc^*^=kFc[1+0.001xFc^2^λ^3^/sin(2θ)]^-1/4^
0 restraints	Extinction coefficient: 0.014 (2)

### Dimethyl 1-phenyl-1H-pyrazole-3,4-dicarboxylate (II) Special details

**Table:**

Geometry. All esds (except the esd in the dihedral angle between two l.s. planes) are estimated using the full covariance matrix. The cell esds are taken into account individually in the estimation of esds in distances, angles and torsion angles; correlations between esds in cell parameters are only used when they are defined by crystal symmetry. An approximate (isotropic) treatment of cell esds is used for estimating esds involving l.s. planes.

### Dimethyl 1-phenyl-1H-pyrazole-3,4-dicarboxylate (II) Fractional atomic coordinates and isotropic or equivalent isotropic displacement parameters (Å^2^)

**Table:**

	*x*	*y*	*z*	*U*_iso_*/*U*_eq_	
N1	0.5660 (3)	0.34915 (11)	0.69867 (11)	0.0412 (4)	
N2	0.4302 (3)	0.27659 (11)	0.71300 (11)	0.0437 (4)	
C3	0.2695 (3)	0.27445 (13)	0.64031 (13)	0.0410 (5)	
C4	0.3003 (3)	0.34570 (14)	0.57864 (13)	0.0416 (5)	
C5	0.4919 (3)	0.39140 (14)	0.61917 (13)	0.0438 (5)	
H5	0.5587	0.4424	0.5959	0.053*	
C11	0.7563 (3)	0.37397 (13)	0.76566 (13)	0.0414 (5)	
C12	0.9342 (3)	0.42372 (15)	0.73977 (15)	0.0490 (5)	
H12	0.9325	0.4396	0.6791	0.059*	
C13	1.1146 (4)	0.44954 (17)	0.80489 (17)	0.0617 (6)	
H13	1.2344	0.4838	0.7880	0.074*	
C14	1.1201 (4)	0.42556 (18)	0.89396 (18)	0.0683 (7)	
H14	1.2442	0.4423	0.9373	0.082*	
C15	0.9413 (4)	0.3766 (2)	0.91897 (17)	0.0738 (8)	
H15	0.9443	0.3608	0.9797	0.089*	
C16	0.7567 (4)	0.35049 (16)	0.85540 (15)	0.0588 (6)	
H16	0.6353	0.3177	0.8728	0.071*	
C31	0.0986 (4)	0.19862 (14)	0.62958 (15)	0.0461 (5)	
O31	0.0528 (3)	0.15354 (14)	0.56252 (12)	0.0900 (7)	
O32	0.0058 (3)	0.18661 (10)	0.70371 (10)	0.0596 (4)	
C32	−0.1630 (4)	0.11423 (17)	0.70126 (19)	0.0701 (7)	
H32A	−0.2925	0.1283	0.6559	0.105*	
H32B	−0.2120	0.1094	0.7597	0.105*	
H32C	−0.0966	0.0569	0.6866	0.105*	
C41	0.1559 (3)	0.37509 (14)	0.49430 (14)	0.0453 (5)	
O41	0.2172 (3)	0.42800 (13)	0.44075 (11)	0.0740 (5)	
O42	−0.0501 (2)	0.33820 (12)	0.48518 (10)	0.0614 (5)	
C42	−0.2056 (4)	0.3587 (2)	0.40269 (17)	0.0704 (7)	
H42A	−0.2395	0.4233	0.4004	0.106*	
H42B	−0.3449	0.3244	0.4020	0.106*	
H42C	−0.1355	0.3419	0.3509	0.106*	

### Dimethyl 1-phenyl-1H-pyrazole-3,4-dicarboxylate (II) Atomic displacement parameters (Å^2^)

**Table:**

	*U*^11^	*U*^22^	*U*^33^	*U*^12^	*U*^13^	*U*^23^
N1	0.0431 (9)	0.0388 (9)	0.0406 (10)	−0.0017 (7)	0.0024 (7)	0.0039 (7)
N2	0.0470 (9)	0.0380 (9)	0.0449 (10)	−0.0039 (7)	0.0027 (8)	0.0052 (7)
C3	0.0437 (11)	0.0410 (11)	0.0379 (11)	−0.0009 (9)	0.0049 (9)	−0.0003 (9)
C4	0.0433 (11)	0.0430 (11)	0.0382 (11)	0.0000 (9)	0.0055 (9)	0.0021 (9)
C5	0.0456 (11)	0.0450 (11)	0.0407 (11)	−0.0029 (9)	0.0065 (9)	0.0057 (9)
C11	0.0408 (10)	0.0389 (11)	0.0425 (12)	0.0041 (9)	−0.0002 (9)	−0.0004 (9)
C12	0.0407 (11)	0.0571 (13)	0.0493 (12)	0.0034 (10)	0.0069 (10)	−0.0016 (10)
C13	0.0429 (12)	0.0712 (16)	0.0695 (17)	−0.0039 (11)	0.0043 (12)	−0.0028 (13)
C14	0.0550 (14)	0.0788 (18)	0.0641 (17)	−0.0031 (13)	−0.0130 (12)	−0.0067 (14)
C15	0.0765 (17)	0.091 (2)	0.0477 (15)	−0.0081 (15)	−0.0110 (13)	0.0077 (13)
C16	0.0609 (14)	0.0625 (15)	0.0500 (14)	−0.0100 (11)	−0.0010 (11)	0.0084 (11)
C31	0.0522 (12)	0.0398 (11)	0.0445 (12)	−0.0027 (9)	0.0018 (10)	−0.0008 (9)
O31	0.1229 (16)	0.0839 (13)	0.0665 (12)	−0.0478 (12)	0.0247 (11)	−0.0260 (10)
O32	0.0649 (10)	0.0556 (9)	0.0613 (10)	−0.0205 (8)	0.0191 (8)	−0.0043 (8)
C32	0.0586 (14)	0.0551 (15)	0.100 (2)	−0.0169 (11)	0.0222 (14)	0.0029 (14)
C41	0.0473 (11)	0.0485 (12)	0.0398 (11)	−0.0036 (10)	0.0057 (9)	0.0000 (9)
O41	0.0718 (11)	0.0869 (13)	0.0584 (10)	−0.0226 (9)	−0.0054 (8)	0.0310 (9)
O42	0.0476 (9)	0.0812 (11)	0.0519 (10)	−0.0100 (8)	−0.0034 (7)	0.0123 (8)
C42	0.0536 (14)	0.0910 (19)	0.0593 (16)	0.0017 (13)	−0.0144 (12)	0.0035 (13)

### Dimethyl 1-phenyl-1H-pyrazole-3,4-dicarboxylate (II) Geometric parameters (Å, º)

**Table:**

N1—C5	1.344 (2)	C14—H14	0.9300
N1—N2	1.360 (2)	C15—C16	1.381 (3)
N1—C11	1.428 (2)	C15—H15	0.9300
N2—C3	1.324 (2)	C16—H16	0.9300
C3—C4	1.413 (3)	C31—O31	1.189 (2)
C3—C31	1.485 (3)	C31—O32	1.316 (2)
C4—C5	1.368 (3)	O32—C32	1.445 (3)
C4—C41	1.467 (3)	C32—H32A	0.9600
C5—H5	0.9300	C32—H32B	0.9600
C11—C16	1.377 (3)	C32—H32C	0.9600
C11—C12	1.377 (3)	C41—O41	1.202 (2)
C12—C13	1.376 (3)	C41—O42	1.316 (2)
C12—H12	0.9300	O42—C42	1.445 (3)
C13—C14	1.365 (3)	C42—H42A	0.9600
C13—H13	0.9300	C42—H42B	0.9600
C14—C15	1.370 (4)	C42—H42C	0.9600
			
C5—N1—N2	111.86 (15)	C14—C15—H15	119.5
C5—N1—C11	127.77 (16)	C16—C15—H15	119.5
N2—N1—C11	120.34 (15)	C11—C16—C15	118.6 (2)
C3—N2—N1	104.78 (15)	C11—C16—H16	120.7
N2—C3—C4	111.42 (17)	C15—C16—H16	120.7
N2—C3—C31	119.60 (17)	O31—C31—O32	124.04 (19)
C4—C3—C31	128.80 (18)	O31—C31—C3	124.1 (2)
C5—C4—C3	104.46 (17)	O32—C31—C3	111.82 (17)
C5—C4—C41	124.54 (18)	C31—O32—C32	116.71 (18)
C3—C4—C41	130.71 (18)	O32—C32—H32A	109.5
N1—C5—C4	107.48 (17)	O32—C32—H32B	109.5
N1—C5—H5	126.3	H32A—C32—H32B	109.5
C4—C5—H5	126.3	O32—C32—H32C	109.5
C16—C11—C12	120.88 (19)	H32A—C32—H32C	109.5
C16—C11—N1	119.82 (18)	H32B—C32—H32C	109.5
C12—C11—N1	119.27 (18)	O41—C41—O42	124.03 (19)
C13—C12—C11	119.1 (2)	O41—C41—C4	123.92 (19)
C13—C12—H12	120.5	O42—C41—C4	112.03 (17)
C11—C12—H12	120.5	C41—O42—C42	117.33 (18)
C14—C13—C12	120.9 (2)	O42—C42—H42A	109.5
C14—C13—H13	119.5	O42—C42—H42B	109.5
C12—C13—H13	119.5	H42A—C42—H42B	109.5
C13—C14—C15	119.4 (2)	O42—C42—H42C	109.5
C13—C14—H14	120.3	H42A—C42—H42C	109.5
C15—C14—H14	120.3	H42B—C42—H42C	109.5
C14—C15—C16	121.0 (2)		
			
C5—N1—N2—C3	0.1 (2)	C11—C12—C13—C14	0.8 (3)
C11—N1—N2—C3	178.19 (16)	C12—C13—C14—C15	−1.3 (4)
N1—N2—C3—C4	−0.1 (2)	C13—C14—C15—C16	0.6 (4)
N1—N2—C3—C31	175.56 (16)	C12—C11—C16—C15	−1.0 (3)
N2—C3—C4—C5	0.0 (2)	N1—C11—C16—C15	−179.0 (2)
C31—C3—C4—C5	−175.11 (19)	C14—C15—C16—C11	0.5 (4)
N2—C3—C4—C41	−173.87 (19)	N2—C3—C31—O31	−130.0 (2)
C31—C3—C4—C41	11.0 (3)	C4—C3—C31—O31	44.8 (3)
N2—N1—C5—C4	−0.1 (2)	N2—C3—C31—O32	49.3 (2)
C11—N1—C5—C4	−177.99 (17)	C4—C3—C31—O32	−135.9 (2)
C3—C4—C5—N1	0.0 (2)	O31—C31—O32—C32	−0.6 (3)
C41—C4—C5—N1	174.43 (18)	C3—C31—O32—C32	−179.80 (17)
C5—N1—C11—C16	153.3 (2)	C5—C4—C41—O41	18.7 (3)
N2—N1—C11—C16	−24.4 (3)	C3—C4—C41—O41	−168.5 (2)
C5—N1—C11—C12	−24.7 (3)	C5—C4—C41—O42	−160.01 (19)
N2—N1—C11—C12	157.57 (17)	C3—C4—C41—O42	12.8 (3)
C16—C11—C12—C13	0.3 (3)	O41—C41—O42—C42	4.0 (3)
N1—C11—C12—C13	178.35 (18)	C4—C41—O42—C42	−177.34 (18)

### Dimethyl 1-phenyl-1H-pyrazole-3,4-dicarboxylate (II) Hydrogen-bond geometry (Å, º)

**Table:**

*D*—H···*A*	*D*—H	H···*A*	*D*···*A*	*D*—H···*A*
C5—H5···O41^i^	0.93	2.41	3.331 (3)	170

Symmetry code: (i) −*x*+1, −*y*+1, −*z*+1.

### Dimethyl 1-(4-methylphenyl)-1H-pyrazole-3,4-dicarboxylate (III) Crystal data

**Table:**

C_14_H_14_N_2_O_4_	*Z* = 2
*M**_r_* = 274.27	*F*(000) = 288
Triclinic, *P*1	*D*_x_ = 1.329 Mg m^−^^3^
*a* = 7.6546 (5) Å	Mo *K*α radiation, λ = 0.71073 Å
*b* = 8.0959 (5) Å	Cell parameters from 2526 reflections
*c* = 11.3065 (6) Å	θ = 2.6–25.5°
α = 78.988 (3)°	µ = 0.10 mm^−^^1^
β = 85.527 (3)°	*T* = 296 K
γ = 87.548 (4)°	Block, brown
*V* = 685.40 (7) Å^3^	0.16 × 0.15 × 0.12 mm

### Dimethyl 1-(4-methylphenyl)-1H-pyrazole-3,4-dicarboxylate (III) Data collection

**Table:**

Bruker Kappa APEXII CCD diffractometer	2526 independent reflections
Radiation source: fine focus sealed tube	1777 reflections with *I* > 2σ(*I*)
Graphite monochromator	*R*_int_ = 0.030
φ and ω scans	θ_max_ = 25.5°, θ_min_ = 2.6°
Absorption correction: multi-scan (SADABS; Sheldrick, 2008a)	*h* = −9→9
*T*_min_ = 0.955, *T*_max_ = 0.988	*k* = −9→9
12818 measured reflections	*l* = −13→13

### Dimethyl 1-(4-methylphenyl)-1H-pyrazole-3,4-dicarboxylate (III) Refinement

**Table:**

Refinement on *F*^2^	Hydrogen site location: inferred from neighbouring sites
Least-squares matrix: full	H-atom parameters constrained
*R*[*F*^2^ > 2σ(*F*^2^)] = 0.044	*w* = 1/[σ^2^(*F*_o_^2^) + (0.0529*P*)^2^ + 0.2439*P*] where *P* = (*F*_o_^2^ + 2*F*_c_^2^)/3
*wR*(*F*^2^) = 0.128	(Δ/σ)_max_ < 0.001
*S* = 1.05	Δρ_max_ = 0.24 e Å^−^^3^
2526 reflections	Δρ_min_ = −0.24 e Å^−^^3^
196 parameters	Extinction correction: SHELXL2014 (Sheldrick, 2015), Fc^*^=kFc[1+0.001xFc^2^λ^3^/sin(2θ)]^-1/4^
7 restraints	Extinction coefficient: 0.024 (4)

### Dimethyl 1-(4-methylphenyl)-1H-pyrazole-3,4-dicarboxylate (III) Special details

**Table:**

Geometry. All esds (except the esd in the dihedral angle between two l.s. planes) are estimated using the full covariance matrix. The cell esds are taken into account individually in the estimation of esds in distances, angles and torsion angles; correlations between esds in cell parameters are only used when they are defined by crystal symmetry. An approximate (isotropic) treatment of cell esds is used for estimating esds involving l.s. planes.

### Dimethyl 1-(4-methylphenyl)-1H-pyrazole-3,4-dicarboxylate (III) Fractional atomic coordinates and isotropic or equivalent isotropic displacement parameters (Å^2^)

**Table:**

	*x*	*y*	*z*	*U*_iso_*/*U*_eq_	Occ. (<1)
N1	0.3938 (2)	0.7228 (2)	0.38159 (13)	0.0446 (4)	
N2	0.4458 (2)	0.7773 (2)	0.26297 (14)	0.0477 (4)	
C3	0.3163 (2)	0.7413 (3)	0.20267 (16)	0.0426 (5)	
C4	0.1791 (2)	0.6623 (2)	0.28127 (16)	0.0428 (5)	
C5	0.2362 (3)	0.6525 (3)	0.39480 (17)	0.0464 (5)	
H5	0.1765	0.6057	0.4675	0.056*	
C11	0.5036 (3)	0.7482 (3)	0.47287 (17)	0.0446 (5)	
C12	0.4529 (3)	0.6979 (3)	0.59269 (19)	0.0634 (7)	
H12	0.3468	0.6450	0.6157	0.076*	
C13	0.5596 (3)	0.7261 (3)	0.6790 (2)	0.0663 (7)	
H13	0.5240	0.6911	0.7602	0.080*	
C14	0.7168 (3)	0.8041 (3)	0.6491 (2)	0.0567 (6)	
C15	0.7647 (3)	0.8500 (4)	0.5289 (2)	0.0758 (8)	
H15	0.8717	0.9011	0.5059	0.091*	
C16	0.6612 (3)	0.8238 (3)	0.4403 (2)	0.0686 (7)	
H16	0.6979	0.8572	0.3592	0.082*	
C141	0.8328 (4)	0.8354 (3)	0.7436 (2)	0.0797 (8)	
H14A	0.7677	0.8190	0.8208	0.119*	
H14B	0.8726	0.9489	0.7233	0.119*	
H14C	0.9319	0.7584	0.7470	0.119*	
C31A	0.3342 (3)	0.7907 (3)	0.06874 (18)	0.0505 (5)	0.71 (2)
O31A	0.4587 (11)	0.7554 (17)	0.0048 (5)	0.093 (3)	0.71 (2)
O32A	0.2044 (10)	0.8936 (9)	0.0288 (6)	0.0542 (14)	0.71 (2)
C32A	0.2113 (19)	0.9575 (17)	−0.0998 (7)	0.0653 (16)	0.71 (2)
H32A	0.2195	0.8650	−0.1421	0.098*	0.71 (2)
H32B	0.3121	1.0261	−0.1233	0.098*	0.71 (2)
H32C	0.1070	1.0239	−0.1196	0.098*	0.71 (2)
C31B	0.3342 (3)	0.7907 (3)	0.06874 (18)	0.0505 (5)	0.29 (2)
O31B	0.413 (2)	0.6979 (19)	0.0113 (14)	0.093 (3)	0.29 (2)
O32B	0.233 (2)	0.9199 (19)	0.0250 (15)	0.0542 (14)	0.29 (2)
C32B	0.234 (5)	0.966 (4)	−0.1050 (17)	0.0653 (16)	0.29 (2)
H32D	0.1476	0.9035	−0.1334	0.098*	0.29 (2)
H32E	0.3479	0.9404	−0.1404	0.098*	0.29 (2)
H32F	0.2080	1.0842	−0.1275	0.098*	0.29 (2)
C41	0.0150 (3)	0.5929 (3)	0.25809 (17)	0.0456 (5)	
O41	−0.0860 (2)	0.5221 (2)	0.33564 (14)	0.0756 (6)	
O42	−0.01086 (18)	0.61505 (19)	0.14123 (12)	0.0550 (4)	
C42	−0.1738 (3)	0.5551 (3)	0.1112 (2)	0.0679 (7)	
H42A	−0.2701	0.6035	0.1538	0.102*	
H42B	−0.1748	0.4346	0.1339	0.102*	
H42C	−0.1847	0.5873	0.0258	0.102*	

### Dimethyl 1-(4-methylphenyl)-1H-pyrazole-3,4-dicarboxylate (III) Atomic displacement parameters (Å^2^)

**Table:**

	*U*^11^	*U*^22^	*U*^33^	*U*^12^	*U*^13^	*U*^23^
N1	0.0440 (9)	0.0561 (11)	0.0331 (8)	−0.0093 (8)	−0.0050 (7)	−0.0041 (7)
N2	0.0458 (10)	0.0613 (11)	0.0345 (9)	−0.0105 (8)	−0.0028 (7)	−0.0030 (8)
C3	0.0397 (11)	0.0511 (12)	0.0369 (10)	−0.0032 (9)	−0.0047 (8)	−0.0067 (9)
C4	0.0407 (10)	0.0505 (12)	0.0372 (10)	−0.0050 (9)	−0.0045 (8)	−0.0063 (9)
C5	0.0436 (11)	0.0567 (13)	0.0373 (10)	−0.0110 (10)	−0.0009 (8)	−0.0036 (9)
C11	0.0449 (11)	0.0505 (12)	0.0390 (11)	−0.0044 (9)	−0.0084 (8)	−0.0073 (9)
C12	0.0539 (13)	0.0937 (19)	0.0424 (12)	−0.0174 (12)	−0.0051 (10)	−0.0085 (12)
C13	0.0673 (16)	0.0924 (19)	0.0403 (12)	−0.0055 (14)	−0.0108 (11)	−0.0117 (12)
C14	0.0644 (15)	0.0543 (13)	0.0555 (14)	0.0014 (11)	−0.0219 (11)	−0.0142 (10)
C15	0.0671 (16)	0.097 (2)	0.0649 (16)	−0.0367 (14)	−0.0163 (12)	−0.0063 (14)
C16	0.0661 (15)	0.0951 (19)	0.0441 (12)	−0.0334 (14)	−0.0082 (11)	−0.0035 (12)
C141	0.092 (2)	0.0787 (19)	0.0782 (18)	0.0009 (15)	−0.0441 (15)	−0.0260 (14)
C31A	0.0409 (11)	0.0727 (15)	0.0380 (11)	−0.0064 (11)	−0.0031 (9)	−0.0093 (10)
O31A	0.070 (3)	0.159 (6)	0.0434 (12)	0.035 (4)	0.0031 (17)	−0.010 (2)
O32A	0.054 (2)	0.062 (2)	0.0395 (9)	−0.008 (2)	0.0009 (13)	0.0060 (12)
C32A	0.075 (4)	0.0739 (19)	0.0406 (14)	−0.017 (3)	−0.0089 (17)	0.0103 (13)
C31B	0.0409 (11)	0.0727 (15)	0.0380 (11)	−0.0064 (11)	−0.0031 (9)	−0.0093 (10)
O31B	0.070 (3)	0.159 (6)	0.0434 (12)	0.035 (4)	0.0031 (17)	−0.010 (2)
O32B	0.054 (2)	0.062 (2)	0.0395 (9)	−0.008 (2)	0.0009 (13)	0.0060 (12)
C32B	0.075 (4)	0.0739 (19)	0.0406 (14)	−0.017 (3)	−0.0089 (17)	0.0103 (13)
C41	0.0421 (11)	0.0557 (13)	0.0387 (11)	−0.0047 (10)	−0.0025 (9)	−0.0077 (9)
O41	0.0581 (10)	0.1211 (15)	0.0449 (9)	−0.0394 (10)	0.0017 (7)	−0.0031 (9)
O42	0.0519 (9)	0.0729 (10)	0.0403 (8)	−0.0177 (7)	−0.0088 (6)	−0.0048 (7)
C42	0.0550 (14)	0.0942 (19)	0.0590 (14)	−0.0187 (13)	−0.0173 (11)	−0.0169 (13)

### Dimethyl 1-(4-methylphenyl)-1H-pyrazole-3,4-dicarboxylate (III) Geometric parameters (Å, º)

**Table:**

N1—C5	1.341 (2)	C141—H14A	0.9600
N1—N2	1.363 (2)	C141—H14B	0.9600
N1—C11	1.430 (2)	C141—H14C	0.9600
N2—C3	1.319 (2)	C31A—O31A	1.209 (4)
C3—C4	1.413 (3)	C31A—O32A	1.319 (3)
C3—C31A	1.487 (3)	O32A—C32A	1.446 (3)
C4—C5	1.375 (3)	C32A—H32A	0.9600
C4—C41	1.459 (3)	C32A—H32B	0.9600
C5—H5	0.9300	C32A—H32C	0.9600
C11—C12	1.368 (3)	O32B—C32B	1.445 (5)
C11—C16	1.369 (3)	C32B—H32D	0.9600
C12—C13	1.378 (3)	C32B—H32E	0.9600
C12—H12	0.9300	C32B—H32F	0.9600
C13—C14	1.372 (3)	C41—O41	1.198 (2)
C13—H13	0.9300	C41—O42	1.328 (2)
C14—C15	1.363 (3)	O42—C42	1.445 (2)
C14—C141	1.505 (3)	C42—H42A	0.9600
C15—C16	1.377 (3)	C42—H42B	0.9600
C15—H15	0.9300	C42—H42C	0.9600
C16—H16	0.9300		
			
C5—N1—N2	111.67 (15)	C14—C141—H14B	109.5
C5—N1—C11	128.79 (16)	H14A—C141—H14B	109.5
N2—N1—C11	119.52 (15)	C14—C141—H14C	109.5
C3—N2—N1	105.05 (15)	H14A—C141—H14C	109.5
N2—C3—C4	111.50 (16)	H14B—C141—H14C	109.5
N2—C3—C31A	117.41 (17)	O31A—C31A—O32A	123.5 (3)
C4—C3—C31A	131.07 (17)	O31A—C31A—C3	125.0 (3)
C5—C4—C3	104.27 (16)	O32A—C31A—C3	111.1 (3)
C5—C4—C41	123.78 (17)	C31A—O32A—C32A	116.5 (3)
C3—C4—C41	131.86 (17)	O32A—C32A—H32A	109.5
N1—C5—C4	107.50 (17)	O32A—C32A—H32B	109.5
N1—C5—H5	126.2	H32A—C32A—H32B	109.5
C4—C5—H5	126.2	O32A—C32A—H32C	109.5
C12—C11—C16	119.48 (19)	H32A—C32A—H32C	109.5
C12—C11—N1	120.75 (18)	H32B—C32A—H32C	109.5
C16—C11—N1	119.77 (18)	O32B—C32B—H32D	109.5
C11—C12—C13	119.7 (2)	O32B—C32B—H32E	109.5
C11—C12—H12	120.1	H32D—C32B—H32E	109.5
C13—C12—H12	120.1	O32B—C32B—H32F	109.5
C14—C13—C12	122.1 (2)	H32D—C32B—H32F	109.5
C14—C13—H13	119.0	H32E—C32B—H32F	109.5
C12—C13—H13	119.0	O41—C41—O42	123.08 (18)
C15—C14—C13	116.7 (2)	O41—C41—C4	123.97 (18)
C15—C14—C141	121.4 (2)	O42—C41—C4	112.95 (17)
C13—C14—C141	121.9 (2)	C41—O42—C42	116.23 (16)
C14—C15—C16	122.7 (2)	O42—C42—H42A	109.5
C14—C15—H15	118.6	O42—C42—H42B	109.5
C16—C15—H15	118.6	H42A—C42—H42B	109.5
C11—C16—C15	119.3 (2)	O42—C42—H42C	109.5
C11—C16—H16	120.4	H42A—C42—H42C	109.5
C15—C16—H16	120.4	H42B—C42—H42C	109.5
C14—C141—H14A	109.5		
			
C5—N1—N2—C3	0.7 (2)	C12—C13—C14—C15	−1.3 (4)
C11—N1—N2—C3	−177.91 (17)	C12—C13—C14—C141	179.7 (2)
N1—N2—C3—C4	−0.2 (2)	C13—C14—C15—C16	1.3 (4)
N1—N2—C3—C31A	178.57 (18)	C141—C14—C15—C16	−179.7 (3)
N2—C3—C4—C5	−0.3 (2)	C12—C11—C16—C15	−0.9 (4)
C31A—C3—C4—C5	−178.9 (2)	N1—C11—C16—C15	179.1 (2)
N2—C3—C4—C41	−176.7 (2)	C14—C15—C16—C11	−0.2 (4)
C31A—C3—C4—C41	4.7 (4)	N2—C3—C31A—O31A	52.4 (9)
N2—N1—C5—C4	−0.9 (2)	C4—C3—C31A—O31A	−129.1 (9)
C11—N1—C5—C4	177.57 (18)	N2—C3—C31A—O32A	−121.0 (5)
C3—C4—C5—N1	0.7 (2)	C4—C3—C31A—O32A	57.5 (6)
C41—C4—C5—N1	177.50 (18)	O31A—C31A—O32A—C32A	3.6 (10)
C5—N1—C11—C12	0.3 (3)	C3—C31A—O32A—C32A	177.1 (8)
N2—N1—C11—C12	178.6 (2)	C5—C4—C41—O41	0.6 (3)
C5—N1—C11—C16	−179.7 (2)	C3—C4—C41—O41	176.5 (2)
N2—N1—C11—C16	−1.4 (3)	C5—C4—C41—O42	−178.86 (19)
C16—C11—C12—C13	0.9 (4)	C3—C4—C41—O42	−3.0 (3)
N1—C11—C12—C13	−179.0 (2)	O41—C41—O42—C42	2.7 (3)
C11—C12—C13—C14	0.2 (4)	C4—C41—O42—C42	−177.82 (18)

### Dimethyl 1-(4-methylphenyl)-1H-pyrazole-3,4-dicarboxylate (III) Hydrogen-bond geometry (Å, º)

**Table:**

*D*—H···*A*	*D*—H	H···*A*	*D*···*A*	*D*—H···*A*
C5—H5···O41^i^	0.93	2.33	3.249 (3)	168
C12—H12···O41^i^	0.93	2.43	3.352 (3)	173

Symmetry code: (i) −*x*, −*y*+1, −*z*+1.

### 1-(4-Methoxyphenyl)-1H-pyrazole-3,4-dicarbohydrazide (IV) Crystal data

**Table:**

C_12_H_14_N_6_O_3_	*F*(000) = 608
*M**_r_* = 290.29	*D*_x_ = 1.492 Mg m^−^^3^
Monoclinic, *P*2_1_/*n*	Mo *K*α radiation, λ = 0.71073 Å
*a* = 7.6030 (6) Å	Cell parameters from 2521 reflections
*b* = 22.6605 (19) Å	θ = 2.9–26.1°
*c* = 7.6751 (7) Å	µ = 0.11 mm^−^^1^
β = 102.284 (3)°	*T* = 296 K
*V* = 1292.05 (19) Å^3^	Block, colourless
*Z* = 4	0.14 × 0.13 × 0.11 mm

### 1-(4-Methoxyphenyl)-1H-pyrazole-3,4-dicarbohydrazide (IV) Data collection

**Table:**

Bruker Kappa APEXII CCD diffractometer	2521 independent reflections
Radiation source: fine focus sealed tube	1722 reflections with *I* > 2σ(*I*)
Graphite monochromator	*R*_int_ = 0.053
φ and ω scans	θ_max_ = 26.1°, θ_min_ = 2.9°
Absorption correction: multi-scan (SADABS; Sheldrick, 2008a)	*h* = −9→9
*T*_min_ = 0.929, *T*_max_ = 0.988	*k* = −27→27
20456 measured reflections	*l* = −9→9

### 1-(4-Methoxyphenyl)-1H-pyrazole-3,4-dicarbohydrazide (IV) Refinement

**Table:**

Refinement on *F*^2^	Hydrogen site location: mixed
Least-squares matrix: full	H atoms treated by a mixture of independent and constrained refinement
*R*[*F*^2^ > 2σ(*F*^2^)] = 0.046	*w* = 1/[σ^2^(*F*_o_^2^) + (0.027*P*)^2^ + 1.1086*P*] where *P* = (*F*_o_^2^ + 2*F*_c_^2^)/3
*wR*(*F*^2^) = 0.108	(Δ/σ)_max_ < 0.001
*S* = 1.05	Δρ_max_ = 0.20 e Å^−^^3^
2521 reflections	Δρ_min_ = −0.21 e Å^−^^3^
210 parameters	Extinction correction: SHELXL2014 (Sheldrick, 2015), Fc^*^=kFc[1+0.001xFc^2^λ^3^/sin(2θ)]^-1/4^
0 restraints	Extinction coefficient: 0.0045 (9)

### 1-(4-Methoxyphenyl)-1H-pyrazole-3,4-dicarbohydrazide (IV) Special details

**Table:**

Geometry. All esds (except the esd in the dihedral angle between two l.s. planes) are estimated using the full covariance matrix. The cell esds are taken into account individually in the estimation of esds in distances, angles and torsion angles; correlations between esds in cell parameters are only used when they are defined by crystal symmetry. An approximate (isotropic) treatment of cell esds is used for estimating esds involving l.s. planes.

### 1-(4-Methoxyphenyl)-1H-pyrazole-3,4-dicarbohydrazide (IV) Fractional atomic coordinates and isotropic or equivalent isotropic displacement parameters (Å^2^)

**Table:**

	*x*	*y*	*z*	*U*_iso_*/*U*_eq_	
N1	0.7660 (2)	0.36969 (8)	0.6437 (2)	0.0283 (4)	
N2	0.7948 (2)	0.32224 (8)	0.5473 (2)	0.0314 (5)	
C3	0.7018 (3)	0.33229 (9)	0.3822 (3)	0.0275 (5)	
C4	0.6115 (3)	0.38765 (9)	0.3717 (3)	0.0280 (5)	
C5	0.6564 (3)	0.40941 (10)	0.5433 (3)	0.0298 (5)	
H5	0.6181	0.4450	0.5828	0.036*	
C11	0.8572 (3)	0.37355 (9)	0.8268 (3)	0.0276 (5)	
C12	0.8449 (3)	0.42341 (10)	0.9244 (3)	0.0344 (6)	
H12	0.7747	0.4550	0.8727	0.041*	
C13	0.9380 (3)	0.42666 (11)	1.1013 (3)	0.0365 (6)	
H13	0.9289	0.4603	1.1681	0.044*	
C14	1.0436 (3)	0.38002 (10)	1.1778 (3)	0.0317 (5)	
C15	1.0555 (3)	0.33004 (10)	1.0777 (3)	0.0368 (6)	
H15	1.1270	0.2986	1.1285	0.044*	
C16	0.9622 (3)	0.32644 (10)	0.9034 (3)	0.0352 (6)	
H16	0.9696	0.2925	0.8372	0.042*	
O14	1.1404 (2)	0.37883 (8)	1.3504 (2)	0.0444 (5)	
C141	1.1307 (4)	0.42931 (12)	1.4580 (3)	0.0493 (7)	
H14A	1.1762	0.4631	1.4063	0.074*	
H14B	1.2016	0.4227	1.5757	0.074*	
H14C	1.0077	0.4362	1.4647	0.074*	
C31	0.7103 (3)	0.28637 (9)	0.2463 (3)	0.0295 (5)	
O31	0.6069 (2)	0.28624 (7)	0.0961 (2)	0.0414 (5)	
N31	0.8329 (3)	0.24513 (8)	0.2954 (3)	0.0356 (5)	
H31	0.911 (3)	0.2477 (11)	0.397 (3)	0.043*	
N32	0.8637 (3)	0.19995 (10)	0.1787 (3)	0.0520 (6)	
H32A	0.782 (4)	0.2047 (12)	0.064 (4)	0.062*	
H32B	0.841 (4)	0.1612 (13)	0.232 (4)	0.062*	
C41	0.5024 (3)	0.42229 (10)	0.2236 (3)	0.0301 (5)	
O41	0.4610 (2)	0.47395 (7)	0.2471 (2)	0.0456 (5)	
N41	0.4562 (3)	0.39535 (9)	0.0672 (2)	0.0358 (5)	
H41	0.500 (3)	0.3563 (12)	0.058 (3)	0.043*	
N42	0.3537 (3)	0.42254 (10)	−0.0869 (3)	0.0435 (6)	
H42A	0.412 (4)	0.4577 (13)	−0.110 (3)	0.052*	
H42B	0.262 (4)	0.4361 (13)	−0.060 (4)	0.052*	

### 1-(4-Methoxyphenyl)-1H-pyrazole-3,4-dicarbohydrazide (IV) Atomic displacement parameters (Å^2^)

**Table:**

	*U*^11^	*U*^22^	*U*^33^	*U*^12^	*U*^13^	*U*^23^
N1	0.0298 (10)	0.0300 (10)	0.0239 (10)	0.0028 (8)	0.0031 (8)	−0.0014 (8)
N2	0.0341 (11)	0.0310 (10)	0.0265 (11)	0.0033 (8)	0.0006 (9)	−0.0032 (8)
C3	0.0260 (12)	0.0287 (11)	0.0264 (12)	−0.0016 (9)	0.0022 (10)	0.0004 (9)
C4	0.0274 (12)	0.0288 (11)	0.0266 (12)	−0.0023 (9)	0.0027 (10)	0.0022 (9)
C5	0.0289 (12)	0.0277 (11)	0.0317 (13)	0.0018 (10)	0.0040 (10)	0.0021 (10)
C11	0.0265 (12)	0.0319 (12)	0.0238 (12)	−0.0003 (9)	0.0040 (10)	0.0010 (9)
C12	0.0356 (14)	0.0342 (13)	0.0306 (13)	0.0061 (11)	0.0006 (11)	0.0018 (10)
C13	0.0401 (14)	0.0369 (13)	0.0316 (14)	0.0014 (11)	0.0055 (11)	−0.0053 (11)
C14	0.0286 (13)	0.0412 (13)	0.0235 (12)	−0.0034 (10)	0.0010 (10)	0.0028 (10)
C15	0.0371 (14)	0.0356 (13)	0.0337 (14)	0.0078 (11)	−0.0011 (11)	0.0040 (11)
C16	0.0387 (14)	0.0325 (12)	0.0325 (13)	0.0055 (11)	0.0035 (11)	−0.0036 (10)
O14	0.0503 (11)	0.0495 (11)	0.0273 (9)	0.0030 (9)	−0.0051 (8)	−0.0007 (8)
C141	0.0546 (18)	0.0587 (18)	0.0313 (14)	−0.0021 (14)	0.0016 (13)	−0.0058 (13)
C31	0.0289 (13)	0.0281 (12)	0.0296 (13)	−0.0025 (10)	0.0018 (10)	0.0001 (10)
O31	0.0433 (10)	0.0387 (10)	0.0340 (10)	0.0029 (8)	−0.0103 (8)	−0.0066 (7)
N31	0.0417 (12)	0.0299 (10)	0.0309 (11)	0.0056 (9)	−0.0018 (9)	−0.0036 (9)
N32	0.0701 (17)	0.0366 (13)	0.0440 (14)	0.0127 (12)	0.0003 (12)	−0.0093 (11)
C41	0.0297 (12)	0.0311 (12)	0.0288 (13)	0.0003 (10)	0.0050 (10)	0.0024 (10)
O41	0.0640 (12)	0.0331 (9)	0.0364 (10)	0.0141 (8)	0.0030 (9)	0.0014 (8)
N41	0.0391 (12)	0.0367 (11)	0.0274 (11)	0.0063 (9)	−0.0022 (9)	0.0022 (9)
N42	0.0436 (14)	0.0509 (14)	0.0314 (12)	0.0039 (11)	−0.0019 (10)	0.0086 (10)

### 1-(4-Methoxyphenyl)-1H-pyrazole-3,4-dicarbohydrazide (IV) Geometric parameters (Å, º)

**Table:**

N1—N2	1.349 (2)	C15—H15	0.9300
N1—C5	1.351 (3)	C16—H16	0.9300
N1—C11	1.431 (3)	O14—C141	1.422 (3)
N2—C3	1.333 (3)	C141—H14A	0.9600
C3—C4	1.424 (3)	C141—H14B	0.9600
C3—C31	1.485 (3)	C141—H14C	0.9600
C4—C5	1.380 (3)	C31—O31	1.249 (3)
C4—C41	1.482 (3)	C31—N31	1.317 (3)
C5—H5	0.9300	N31—N32	1.412 (3)
C11—C12	1.369 (3)	N31—H31	0.87 (3)
C11—C16	1.387 (3)	N32—H32A	0.97 (3)
C12—C13	1.394 (3)	N32—H32B	1.00 (3)
C12—H12	0.9300	C41—O41	1.236 (3)
C13—C14	1.381 (3)	C41—N41	1.325 (3)
C13—H13	0.9300	N41—N42	1.412 (3)
C14—O14	1.371 (3)	N41—H41	0.95 (3)
C14—C15	1.382 (3)	N42—H42A	0.95 (3)
C15—C16	1.377 (3)	N42—H42B	0.83 (3)
			
N2—N1—C5	111.78 (17)	C15—C16—C11	119.8 (2)
N2—N1—C11	119.05 (17)	C15—C16—H16	120.1
C5—N1—C11	129.07 (18)	C11—C16—H16	120.1
C3—N2—N1	105.65 (17)	C14—O14—C141	117.50 (19)
N2—C3—C4	110.88 (19)	O14—C141—H14A	109.5
N2—C3—C31	117.06 (19)	O14—C141—H14B	109.5
C4—C3—C31	132.06 (19)	H14A—C141—H14B	109.5
C5—C4—C3	104.01 (18)	O14—C141—H14C	109.5
C5—C4—C41	121.8 (2)	H14A—C141—H14C	109.5
C3—C4—C41	134.0 (2)	H14B—C141—H14C	109.5
N1—C5—C4	107.67 (19)	O31—C31—N31	122.0 (2)
N1—C5—H5	126.2	O31—C31—C3	122.5 (2)
C4—C5—H5	126.2	N31—C31—C3	115.47 (19)
C12—C11—C16	120.2 (2)	C31—N31—N32	122.4 (2)
C12—C11—N1	120.81 (19)	C31—N31—H31	120.9 (17)
C16—C11—N1	118.97 (19)	N32—N31—H31	116.0 (17)
C11—C12—C13	119.8 (2)	N31—N32—H32A	109.5 (17)
C11—C12—H12	120.1	N31—N32—H32B	108.1 (16)
C13—C12—H12	120.1	H32A—N32—H32B	110 (2)
C14—C13—C12	120.2 (2)	O41—C41—N41	122.8 (2)
C14—C13—H13	119.9	O41—C41—C4	120.6 (2)
C12—C13—H13	119.9	N41—C41—C4	116.59 (19)
O14—C14—C13	124.7 (2)	C41—N41—N42	123.4 (2)
O14—C14—C15	115.8 (2)	C41—N41—H41	118.0 (15)
C13—C14—C15	119.5 (2)	N42—N41—H41	118.6 (15)
C16—C15—C14	120.5 (2)	N41—N42—H42A	109.1 (16)
C16—C15—H15	119.7	N41—N42—H42B	107 (2)
C14—C15—H15	119.7	H42A—N42—H42B	101 (3)
			
C5—N1—N2—C3	0.2 (2)	C12—C13—C14—C15	−0.3 (3)
C11—N1—N2—C3	−176.56 (18)	O14—C14—C15—C16	179.4 (2)
N1—N2—C3—C4	0.2 (2)	C13—C14—C15—C16	−0.4 (4)
N1—N2—C3—C31	179.81 (18)	C14—C15—C16—C11	0.8 (4)
N2—C3—C4—C5	−0.6 (2)	C12—C11—C16—C15	−0.5 (3)
C31—C3—C4—C5	179.9 (2)	N1—C11—C16—C15	178.0 (2)
N2—C3—C4—C41	175.1 (2)	C13—C14—O14—C141	0.1 (3)
C31—C3—C4—C41	−4.4 (4)	C15—C14—O14—C141	−179.6 (2)
N2—N1—C5—C4	−0.6 (2)	N2—C3—C31—O31	168.0 (2)
C11—N1—C5—C4	175.8 (2)	C4—C3—C31—O31	−12.5 (4)
C3—C4—C5—N1	0.7 (2)	N2—C3—C31—N31	−11.1 (3)
C41—C4—C5—N1	−175.66 (19)	C4—C3—C31—N31	168.4 (2)
N2—N1—C11—C12	173.8 (2)	O31—C31—N31—N32	3.9 (4)
C5—N1—C11—C12	−2.3 (3)	C3—C31—N31—N32	−177.0 (2)
N2—N1—C11—C16	−4.7 (3)	C5—C4—C41—O41	6.1 (3)
C5—N1—C11—C16	179.2 (2)	C3—C4—C41—O41	−169.0 (2)
C16—C11—C12—C13	−0.2 (3)	C5—C4—C41—N41	−175.2 (2)
N1—C11—C12—C13	−178.6 (2)	C3—C4—C41—N41	9.8 (4)
C11—C12—C13—C14	0.6 (4)	O41—C41—N41—N42	−0.8 (4)
C12—C13—C14—O14	180.0 (2)	C4—C41—N41—N42	−179.5 (2)

### 1-(4-Methoxyphenyl)-1H-pyrazole-3,4-dicarbohydrazide (IV) Hydrogen-bond geometry (Å, º)

**Table:**

*D*—H···*A*	*D*—H	H···*A*	*D*···*A*	*D*—H···*A*
N31—H31···O31^i^	0.88 (2)	2.04 (2)	2.851 (3)	153 (2)
N32—H32*A*···O14^ii^	0.97 (3)	2.58 (3)	3.256 (3)	127 (2)
N32—H32*B*···N42^i^	1.00 (2)	2.34 (3)	3.317 (3)	165 (2)
N41—H41···O31	0.95 (3)	1.78 (3)	2.714 (3)	166 (2)
N42—H42*A*···O41^iii^	0.95 (3)	2.21 (3)	3.120 (3)	162 (2)
N42—H42*B*···*Cg*1^iv^	0.83 (3)	2.85 (3)	3.442 (3)	130 (2)
C5—H5···O41^v^	0.93	2.40	3.314 (3)	166
C12—H12···O41^v^	0.93	2.44	3.354 (3)	168

Symmetry codes: (i) *x*+1/2, −*y*+1/2, *z*+1/2; (ii) *x*−1/2, −*y*+1/2, *z*−3/2; (iii) −*x*+1, −*y*+1, −*z*; (iv) *x*−1, *y*, *z*−1; (v) −*x*+1, −*y*+1, −*z*+1.
